# Methodological quality of systematic reviews on Chinese herbal medicine: a methodological survey

**DOI:** 10.1186/s12906-022-03529-w

**Published:** 2022-02-23

**Authors:** Andy K. L. Cheung, Charlene H. L. Wong, Leonard Ho, Irene X. Y. Wu, Fiona Y. T. Ke, Vincent C. H. Chung

**Affiliations:** 1 Jockey Club School of Public Health and Primary Care, The Chinese University of Hong Kong, Shatin, Hong Kong; 2 School of Chinese Medicine, The Chinese University of Hong Kong, Shatin, Hong Kong; 3 5/F, Xiangya School of Public Health, Central South University, 238 Shang-Ma-Yuan-Ling Alley, Kai-Fu District, Changsha, Hunan China

**Keywords:** Meta-analysis, Systematic review, Drugs, Chinese herbal, Evidence-based medicine, Review literature as topic, Medicine, Chinese traditional

## Abstract

**Background:**

Systematic reviews (SRs) synthesise the best evidence of effectiveness and safety on Chinese herbal medicine (CHM). Decision-making should be supported by the high-quality evidence of prudently conducted SRs, but the trustworthiness of conclusions may be limited by poor methodological rigour.

**Methods:**

This survey aimed to examine the methodological quality of a representative sample of SRs on CHM published during January 2018 to March 2020. We conducted literature search in Cochrane Database of Systematic Reviews, MEDLINE via Ovid, and EMBASE via Ovid. Eligible SRs must be in Chinese or English with at least one meta-analysis on the treatment effect of any CHM documented in the 2015 Chinese Pharmacopoeia. Two reviewers extracted the bibliographical characteristics of SRs and appraised their methodological quality using AMSTAR 2 (Assessing the Methodological Quality of Systematic Reviews 2). The associations between bibliographical characteristics and methodological quality were investigated using Kruskal-Wallis tests and Spearman’s rank correlation coefficients.

**Results:**

We sampled and appraised one hundred forty-eight SRs. Overall, one (0.7%) was of high methodological quality; zero (0%), four (2.7%), and one-hundred forty-three (96.6%) SRs were of moderate, low, and critically-low quality. Only thirteen SRs (8.8%) provided a pre-defined protocol; none (0%) provided justifications for including particular primary study designs; six (4.1%) conducted a comprehensive literature search; two (1.4%) provided a list of excluded studies; nine (6.1%) undertook meta-analysis with appropriate methods; and seven (4.7%) reported funding sources of included primary studies. Cochrane reviews had higher overall quality than non-Cochrane reviews (*P* < 0.001). SRs with European funding support were less likely to have critically-low quality when compared with their counterparts (*P* = 0.020). SRs conducted by more authors (*r*_*s*_ = 0.23; *P* = 0.006) and published in higher impact factor journals (*r*_*s*_ = 0.20; *P* = 0.044) were associated with higher methodological quality.

**Conclusions:**

Our results indicated that the methodological quality of SRs on CHM is low. Future authors should enhance the methodological quality through registering a priori protocols, justifying selection of study designs, conducting comprehensive literature search, providing a list of excluded studies with rationales, using appropriate method for meta-analyses, and reporting funding sources among primary studies.

**Supplementary Information:**

The online version contains supplementary material available at 10.1186/s12906-022-03529-w.

## Introduction

According to *The World Health Organization Traditional Medicine Strategy 2014–2023,* the World Health Organization (WHO) advocates evidence-based use of traditional, complementary, and integrative medicine (TCIM) in clinical practice [1]. In Western countries, attempts in integrating TCIM into the healthcare system under an evidence-based approach has been observed in the United States [2] and Australia [3]. In ethnic Chinese societies, Chinese medicine (CM) is considered as the major form of TCIM which constitutes as an important part of the healthcare ecology [4]. CM has been integrated as a part of modern healthcare delivery system in China [5], Taiwan [6] and Hong Kong [7]. In China, 90% of the hospitals have established CM departments, with an annually CM services volume of more than 210 million patients [8]. In Taiwan, a significant upward trend of CM utilisation in the past decade is demonstrated in a population-based cohort study [9]. Overall, number of CM consultation increased by approximately 18% from 2000 to 2010, which accounted for about 29% of total medical visits in Taiwan annually. In Australia, about 20% of the population consume CM services annually [3]. Half of the CM practitioners in Australia reported that Chinese herbal medicine (CHM) is frequently used in clinical practice [10]. Since 2000s, statutory regulation of CM practitioner has been introduced in Australia [11] and Canada [12]. These imply that CM has received substantial attention among both ethnic Chinese societies and Western countries.

According to recently published policy documents of *The Construction Plan for the Chinese Medicine Highlands in the Guangdong-Hong Kong-Macao Greater Bay Area (2020-2025)* [13] and *The Opinions of the Communist Party of China Central Committee and the State Council on Promoting the Preservation, Innovation, and Development of Traditional Chinese Medicine* [14], the Chinese Government has positioned the evidence-based approach as a key direction for CM development. As CHM is one of the most common CM modalities [15], there is an urgency to confirm or refute the effectiveness of CHM via timely evidence synthesis [16]. Although the number of systematic reviews (SRs) on CHM has been increasing [17], a methodological evaluation illustrated that the rigour of SRs on CHM published during 1993–2013 was poor [18]. Methodological flaws might give rise to overestimation or underestimation of pooled intervention effect, and in turn, mislead clinical decision making by biased conclusions [19]. While the application of evidence-based medicine concept and methods in Chinese medicine has been progressing in the past few years [20], it is unclear whether methodological improvements on SR conduct were made since the last evaluation.

This survey aimed to (i) describe the bibliographical characteristics of recent (2018–early 2020) SRs on CHM trials; and (ii) evaluate the methodological quality of recent SRs on CHM trials with AMSTAR 2 (Assessing the Methodological Quality of Systematic Reviews 2) tool. This will inform policy makers and clinicians on whether recent SRs on CHM have improved rigour and are methodologically sufficient for guiding clinical decision-making.

## Methods

### Eligibility criteria

SRs published during January 2018 to March 2020 in Chinese or English were eligible. They must include at least one meta-analysis (MA) on the treatment effect of any CHM. Currently, AMSTAR 2 is yet to incorporate methodological expectations listed in the Synthesis Without Meta-analysis (SWiM) guideline published in 2020 [21]. Therefore, we believe that using the current AMSTAR 2 for appraising SRs without MA may not be ideal, and hence we decided to appraise SR with MA only in this survey. CHM evaluated must be documented in the 2015 Pharmacopoeia of the People’s Republic of China [22]. No restrictions were applied on the dosage forms or route of delivery of CHM. SRs of etiological or diagnostic research, overviews of SRs, conference abstracts, narrative reviews, protocols, and network MAs were excluded. For the duplicates of a SR, only the most up-to-date version was included.

### Literature search

A comprehensive literature search was conducted in three international electronic databases for a representative sample of SRs: (i) Cochrane Database of Systematic Reviews, (ii) MEDLINE via Ovid, and (iii) EMBASE via Ovid. This enabled us to sample both relevant Cochrane and non-Cochrane SRs. Validated search filters for SRs were applied to maximise specificity of search on MEDLINE [23] and EMBASE [24]. One of the authors (Chung), who has had fifteen years of experience in conducting SRs, was responsible for constructing the search strategy adopted in this survey. Detailed search strategies were indexed in Table S1, Supplementary file 1. To ensure representativeness of this methodological survey, we included all SRs so long as they were identified in the search using the validated search filters. This census-like sampling procedure enabled the exploration of a representative sample of SRs which are most utilised by clinicians and policy makers. The adoption of these databases for identifying SRs has been recommended in the Comprehensive Framework of Methods for Conducting, Interpreting and Reporting Overviews [25].

### Literature selection and data extraction

All relevant SRs citations were imported into EndNote 20 (Clarivate Analytics, Philadelphia, Pennsylvania, United States) for de-duplication. Titles and abstracts of retrieved citations were then screened according to the eligibility criteria. Full-text of potentially eligible publications was retrieved for further assessment. We also requested for additional information from the original authors to reaffirm the SRs’ eligibility whenever necessary. Data on bibliographical characteristics of included SRs were extracted using a pre-specified data extraction form. The pre-specified data extraction form has been applied in previous assessments of SRs [18, 26–29] and details are shown in Table S2, Supplementary file 1.

Literature selection and data extraction were done in duplicate by two independent reviewers (Cheung and Ho). Discrepancies were resolved by the discussion between reviewers. Consultation with a third senior investigator (Chung) was undertaken to reach consensus on persistent disagreement.

### Methodological quality assessment

Methodological quality of SRs was critically appraised with AMSTAR 2 [30], a validated appraisal instrument for SRs [31]. AMSTAR 2 has been widely applied in assessing the methodological quality of SRs, for instance in the area of pain relief [32], obesity management [33], Parkinson’s disease [34] and cancer [35]. Each SR was evaluated across sixteen domains based on relevant domain-specific items in AMSTAR 2, and subsequently, an overall rating on methodological quality was achieved [30]. Among the sixteen domains, the following seven are considered as critical domains in influencing the methodological quality [30].

1. Protocol registered before commencement of the review (item 2)
2. Adequacy of the literature search (item 4)
3. Justification for excluding individual studies (item 7)
4. Risk of bias from individual studies being included in the review (item 9)
5. Appropriateness of meta-analytical methods (item 11)
6. Consideration of risk of bias when interpreting the results of the review (item 13)
7. Assessment of presence and likely impact of publication bias (item 15)

Overall rating of the methodological quality is classified as: “high”, “moderate”, “low” and “critically-low” [30].

Methodological quality assessment was performed by two independent reviewers (Cheung and Ho). Discrepancies were resolved by consensus between reviewers. If disagreement persists, it was resolved by consulting a third senior investigator (Wong).

### Data analysis

Data on bibliographical characteristics, and AMSTAR 2 methodological quality assessment of SRs were presented with descriptive statistics. Categorical variables were summarised as frequencies with percentages. Continuous variables were described as medians with ranges or frequencies with means and standard deviation, as appropriate. Kruskal-Wallis tests and Spearman’s rank correlation coefficients were applied to examine the differences in the overall methodological quality across categorical and continuous bibliographic characteristics, respectively. A *p*-value of < 0.05 was considered as statistically significant. All statistical analyses were performed using IBM Statistical Package for Social Sciences version 25.0 software (IBM Corporation, Armonk, New York, United States).

## Results

### Literature screening and selection

**Fig. 1:**
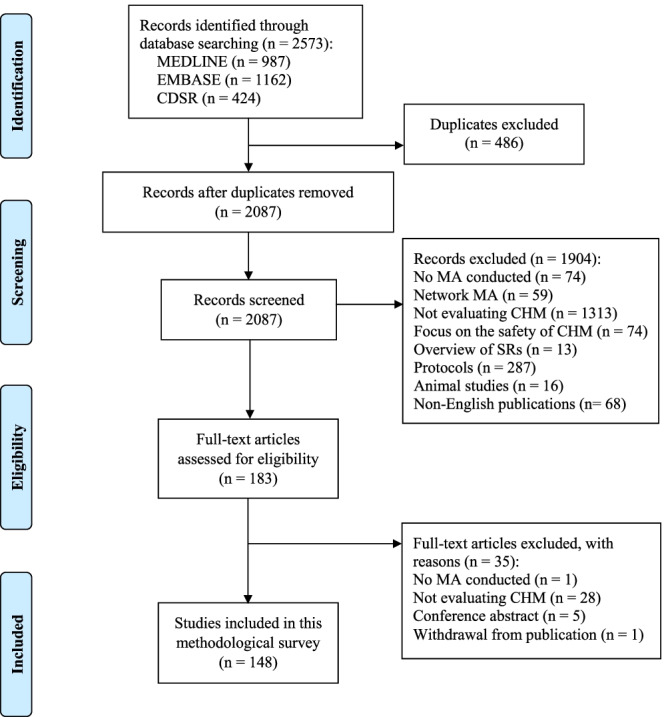
Screening and selection process of SRs on CHM trials from January 2018 to March 2020. *Keys:* CDSR: Cochrane Database of Systematic Reviews. MA: Meta-analysis. CHM: Chinese herbal medicine. SR: Systematic review

A total of 2573 records were retrieved through the database search, 486 duplicates were excluded and 1904 publications were removed after screening of titles and abstracts. Another thirty-five publications were excluded after full-text assessments. Finally, 148 SRs which met the eligibility criteria were included in this study with their bibliographical details (Table S3 and Table S4, Supplementary file 1). Details on literature selection are illustrated in Fig. 1. A full list of excluded SRs is provided in Table S5, Supplementary file 1.

### Bibliographical characteristics of included systematic reviews

The 148 SRs synthesised results from 3022 randomised controlled trials (RCTs), with 288,351 participants involved. Non-Cochrane reviews accounted for the vast majority of SRs (146; 98.6%), and among them, only twelve (8.1%) were updates of prior SRs. The median impact factor of the journal publishing the included SRs was 2.03 (range from 0 to 7.76). A median of sixteen trials were included in each SR (range from 2 to 121). One-hundred and thirty-nine (93.9%) SRs considered the harms of CHM. One-hundred and four (70.3%) SRs had their result of the first primary outcome in favour of CHM intervention (s) with reservation.

The median number of review authors was six (range from 1 to 14), with the highest proportion of corresponding authors being based in Asia (141; 95.3%), followed by Oceania (5; 3.4%) and Europe (2; 1.4%). Most funding sources were originated from Asia (97; 65.5%). Twenty (15.9%) SRs received no funding support. Sources of funding were not declared in twenty-two (14.7%) SRs.

One-hundred and forty-five (98.6%) SRs searched English electronic databases while 139 (93.9%) SRs searched non-English electronic databases. Nevertheless, the language of eligible trials was not reported in 104 (70.3%) SRs. Sixteen (10.8%) SRs included both English and non-English primary studies. One (0.7%) SR included RCTs publishing in English language only while twenty-seven (18.2%) SRs included RCTs publishing only in non-English language. Nearly all SRs (147; 99.3%) included a PRISMA (Preferred Reporting Items for Systematic Reviews and Meta-analysis) -like flow diagram. A large proportion of SRs (111; 75.0%) reported the complete year span of search but only twenty-four (16.2%) SRs reported the full Boolean search terms. The majority of SRs (130; 87.8%) examined risk of bias among included trials by using the Cochrane risk of bias tool, fourteen (9.5%) used the Jadad scale, and only two (1.4%) SRs did not adopt any tool for risk of bias assessment. Detailed records for bibliographical characteristics are shown in Table 1.

**Table 1:** Bibliographical characteristics of 148 systematic reviews on Chinese herbal medicine

Bibliographical characteristics	Results^a^
**Cochrane review**	2 (1.4)
**Non-Cochrane review**	146 (98.6)
**An update of previous SR**	12 (8.1)
An update of previous Cochrane review	0 (0)
An update of a previous non-Cochrane review	12 (8.1)
**Publication year median (range)**	2019 (2018-2020)
**Publication journal impact factor median (range)**	2.03 (0-7.76)
**Number of review authors median (range)**	6 (1-14)
**Location of corresponding author**	
Europe	2 (1.4)
Asia	141 (95.3)
Oceania	5 (3.4)
**Total number of included primary studies**	3022
**Median number of included primary studies in each SR (range)**	16 (2-121)
**Total number of participants included in primary studies**	288,351
**Median number of participants included in primary studies (range)**	1448.50 (100-11,732)
**SRs reporting intervention harms**	139 (93.9)
**Result of the first primary outcome of the SR**	
No significant difference between CHM intervention and control	12 (8.1)
In favour of CHM intervention	32 (21.6)
In favour of CHM intervention with reservation	104 (70.3)
**Funding location of the SR**	
Europe	6 (4.1)
Asia	97 (65.5)
Oceania	1 (0.7)
Not reported	22 (14.9)
No funding support	20 (13.5)
Multiple funding locations	2 (1.3)
**Source of funding, if reported**	
For-profit	1 (0.8)
Not-for-profit	105 (83.3)
No funding support	20 (15.9)
**SRs that searched English databases**	145 (98.0)
**SRs that searched non-English databases**	139 (93.9)
**Report year span of search**	
Yes, reported both starting and ending years	111 (75.0)
Partially, only reported starting years	29 (19.6)
Not mentioned	8 (5.4)
**Search terms reported for one or more electronic databases**	
Topics/free text/keywords/MeSH	98 (66.2)
Full Boolean	24 (16.2)
Readers are referred elsewhere for full search strategy	21 (14.2)
No research term	5 (3.4)
**Eligibility criteria based on language of publication**	
English only	1 (0.7)
Language other than English	27 (18.2)
English and other languages	16 (10.8)
Not reported	104 (70.3)
**Risk of bias assessment tools**	
Cochrane risk of bias tool	130 (87.8)
Jadad scale	14 (9.5)
CONSORT 2010	1 (0.7)
Newcastle-Ottawa Scale	1 (0.7)
Tool not used	2 (1.4)
**Included a PRISMA-like flow diagram**	147 (99.3)

*Keys: SR* systematic review, *MeSH* National Library of Medical Subject Headings, *CHM* Chinese herbal medicine, *CONSORT* CONsolidated Standards of Reporting Trials, *PRISMA* Preferred Reporting Items for Systematic Reviews and Meta-analysis

^a^Values are n (%), or median (range)

### Methodological quality of systematic reviews on Chinese herbal medicine

Performances of the included SRs were low across four critical AMSTAR 2 domains, with less than 10% fulfilling these domain-specific items: thirteen (8.8%) reported an a priori protocol and justified deviations from the protocol (item 2); six (4.1%) used a comprehensive literature search strategy (item 4); two (1.4%) provided a list of excluded studies with justifications on the exclusions (item 7); and nine (6.1%) used appropriate pooling method in MA (item 11).

The SRs performed relatively better across the following three critical AMSTAR 2 domains: one hundred and thirty (87.8%) SRs assessed the risk of bias of the primary studies appropriately (item 9); one-hundred and sixteen (78.4%) accounted for the risk of bias in primary studies when drawing conclusion (item 13); and 89 (60.1%) investigated presence of publication bias (item 15).

Performances were also poor across most of the non-critical AMSTAR 2 domains, with less than 10% of SRs satisfying the following domain-specific items: none of the SRs (0%) elaborated eligibility for selected study design (item 3); and only seven (4.7%) reported sources of funding among primary studies (item 10). Nevertheless, over 80% of SRs had satisfactory performance in three non-critical domains: all SRs (100%) included PICO (population, intervention, comparison, outcome) components in their research questions and inclusion criteria (item 1); one-hundred and twenty-three (83.1%) provided satisfactory explanation along with discussion on observed heterogeneity (item 14); one-hundred and forty-four (97.3%) reported the potential sources of conflict of interest (item 16). Details are presented in Table 2.

**Table 2:** Methodological quality of 148 included systematic reviews on Chinese herbal medicine

AMSTAR 2 items	Yes (%)	Partial Yes (%)	No (%)
1. Did the research questions and inclusion criteria for the review include the components of PICO?	148(100)	NA	0 (0)
2. Did the report of the review contain an explicit statement that the review methods were established prior to the conduct of the review and did the report justify any significant deviations from the protocol?^a^	13 (8.8)	25 (16.9)	110 (74.3)
3. Did the review authors explain their selection of the study designs for inclusion in the review?	0 (0)	NA	148(100)
4. Did the review authors use a comprehensive literature search strategy?^a^	6 (4.1)	136 (91.9)	6 (4.1)
5. Did the review authors perform study selection in duplicate?	96 (64.9)	NA	52 (35.1)
6. Did the review authors perform data extraction in duplicate?	112 (75.7)	NA	36 (24.3)
7. Did the review authors provide a list of excluded studies and justify the exclusions?^a^	2 (1.4)	2 (1.4)	144 (97.3)
8. Did the review authors describe the included studies in adequate detail?	17 (11.5)	117 (79.1)	14 (9.5)
9. Did the review authors use a satisfactory technique for assessing the risk of bias (RoB) in individual studies that were included in the review?^a^	130 (87.8)	0 (0)	18 (12.2)
10. Did the review authors report on the sources of funding for the studies included in the review?	7 (4.7)	NA	141 (95.3)
11. If meta-analysis was performed did the review authors use appropriate methods for statistical combination of results?^a^	9 (6.1)	NA	139 (93.9)
12. If meta-analysis was performed, did the review authors assess the potential impact of RoB in individual studies on the results of the meta-analysis or other evidence synthesis?	30 (20.3)	NA	118 (79.7)
13. Did the review authors account for RoB in individual studies when interpreting/discussing the results of the review?^a^	116 (78.4)	NA	32 (21.6)
14. Did the review authors provide a satisfactory explanation for, and discussion of, any heterogeneity observed in the results of the review?	123 (83.1)	NA	25 (16.9)
15. If they performed quantitative synthesis did the review authors carry out an adequate investigation of publication bias (small study bias) and discuss its likely impact on the results of the review?^a^	89 (60.1)	NA	59 (39.9)
16. Did the review authors report any potential sources of conflict of interest, including any funding they received for conducting the review?	144 (97.3)	NA	4 (2.7)

*Keys: AMSTAR 2* A Measurement Tool to Assess Systematic Reviews 2, *NA* not applicable

^a^Critical domain-specific item that is responsible for rating overall confidence of the review in terms of methodological quality

The overall methodological quality of included SRs on CHM is low. Among the 148 included SRs, only one (0.7%) SR was of high overall methodological quality. The remaining 143 (96.6%) and four (2.7%) SRs were judged as having critically-low and low overall methodological quality, respectively.

### Relationship between bibliographic characteristics and the overall methodological quality of systematic reviews

Results of Kruskal-Wallis tests showed that there were statistically significant differences in overall methodological quality across certain categorical bibliographical characteristics (Table 3). When compared with non-Cochrane reviews, Cochrane reviews were of higher overall quality (0% vs. 50.0%; *P* < 0.001). SRs with funding support from Europe (33.3%) were less likely to be of critically-low quality when compared with those receiving funding from Asia (100%) or Oceania (100%) (*P* = 0.020). Spearman’s rank correlation coefficients indicated that SRs conducted by more authors (*r*_*s*_ = 0.23; *P* = 0.006) and those published in higher impact factor journals (*r*_*s*_ = 0.20; *P* = 0.044) were associated with higher overall methodological quality.

**Table 3:** Overall methodological quality of the included systematic reviews on Chinese herbal medicine by bibliographical characteristics

Characteristics	Critically-low quality^a^	Low quality^a^	Moderate quality^a^	High quality^a^	***P***
**Total**	143 (96.6)	4 (2.7)	0 (0)	1 (0.7)	
**Cochrane Review**					< 0.001^b^
Yes	0 (0)	1 (50.0)	0 (0)	1 (50.0)	
No	143 (97.9)	3 (2.1)	0 (0)	0 (0)	
**An update of a previous SR**					0.797
Yes (Cochrane review)	0 (0)	0 (0)	0 (0)	0 (0)	
Yes (non-Cochrane review)	12 (100)	0 (0)	0 (0)	0 (0)	
No	131 (96.3)	4 (2.9)	0 (0)	1 (0.7)	
**Published year**					0.384
2018	53 (96.4)	1 (1.8)	0 (0)	1 (1.8)	
2019	72 (97.3)	2 (2.7)	0 (0)	0 (0)	
2020	18 (94.7)	1 (5.3)	0 (0)	0 (0)	
**Location of corresponding author**					0.985
Europe	1 (50.0)	1 (50.0)	0 (0)	0 (0)	
Asia	138 (97.9)	2 (1.4)	0 (0)	1 (0.7)	
Oceania	4 (80.0)	1 (20.0)	0 (0)	0 (0)	
**Reported intervention harms**					0.847
Yes	134 (96.4)	4 (2.9)	0 (0)	1 (0.7)	
No	9 (100)	0 (0)	0 (0)	0 (0)	
**Result of the first primary outcome of the SR**					0.233
No significant difference between CHM intervention and control	11 (91.7)	1 (8.3)	0 (0)	0 (0)	
In favour of CHM intervention	30 (93.8)	1 (3.1)	0 (0)	1 (3.1)	
In favour of CHM intervention with reservation	102 (98.1)	2 (1.9)	0 (0)	0 (0)	
**Funding location of the SR**					0.020^b^
Europe	2 (33.3)	3 (50.0)	0 (0)	1 (16.7)	
Asia	97 (100)	0 (0)	0 (0)	0 (0)	
Oceania	1 (100)	0 (0)	0 (0)	0 (0)	
Not reported	22 (100)	0 (0)	0 (0)	0 (0)	
No funding support	20 (100)	0 (0)	0 (0)	0 (0)	
Multiple funding locations	1 (50.0)	1 (50.0)	0 (0)	0 (0)	
**Source of funding, if reported**					0.133
For-profit	0 (0)	1 (100)	0 (0)	0 (0)	
Not-for-profit	101 (96.2)	3 (2.9)	0 (0)	1 (0.9)	
No funding support	20 (100)	0 (0)	0 (0)	0 (0)	
**Searched English databases**					0.948
Yes	140 (96.6)	4 (2.8)	0 (0)	1 (0.7)	
No	3 (100)	0 (0)	0 (0)	0 (0)	
**Searched non-English databases**					0.847
Yes	134 (96.4)	4 (2.9)	0 (0)	1 (0.7)	
No	9 (100)	0 (0)	0 (0)	0 (0)	
**Report year span of search**					0.430
Yes	106 (95.5)	4 (3.6)	0 (0)	1 (0.9)	
Partially	29 (100)	0 (0)	0 (0)	0 (0)	
Not mentioned	8 (100)	0 (0)	0 (0)	0 (0)	
**Search terms reported for one or more electronic databases**					0,500
Topics/free text/keywords/MeSH	97 (99.0)	0 (0)	0 (0)	1 (1.0)	
Full Boolean	22 (91.7)	2 (8.3)	0 (0)	0 (0)	
Readers are referred elsewhere for full search strategy	20 (95.2)	1 (4.8)	0 (0)	0 (0)	
No search term reported	4 (80.0)	1 (20.0)	0 (0)	0 (0)	
**Eligibility criteria based on language of publication**					0.393
English only	1 (100)	0 (0)	0 (0)	0 (0)	
Language other than English	26 (96.3)	1 (3.7)	0 (0)	0 (0)	
English and other languages	14 (87.5)	1 (6.3)	0 (0)	1 (6.3)	
Not reported	102 (98.1)	2 (1.9)	0 (0)	0 (0)	
**Risk of bias assessment tools**					0.701
Cochrane risk of bias tool	125 (96.2)	4 (3.1)	0 (0)	1 (0.8)	
Jadad scale	14 (100)	0 (0)	0 (0)	0 (0)	
CONSORT 2010	1 (100)	0 (0)	0 (0)	0 (0)	
Newcastle-Ottawa Scale	1 (100)	0 (0)	0 (0)	0 (0)	
Tool not used	2 (100)	0 (0)	0 (0)	0 (0)	
**Included a PRISMA-like flow diagram**					0.983
Yes	142 (96.6)	4 (2.7)	0 (0)	1 (0.7)	
No	1 (100)	0 (0)	0 (0)	0 (0)	

*Keys: SR* systematic review, *MeSH* National Library of Medical Subject Headings, *CHM* Chinese herbal medicine, *CONSORT* CONsolidated Standards of Reporting Trials, *PRISMA* Preferred Reporting Items for Systematic Reviews and Meta-analysis

^a^Values are n (% in subgroup)

^b^*P* value of Kruskal-Wallis test was < 0.05

## Discussion

### Summary of results

This survey assessed the methodological quality of a representative sample of 148 SRs on CHM published between 2018 and 2020. Despite all the resources spent in recent years on training and capacity building [17], no improvements in methodological rigour were observed following the evaluation on CHM SRs published during 1993–2013 [18]. In this study, even though Cochrane reviews showed higher overall methodological quality, methodological rigour of most included SRs in this field was low. More than 96% of SRs were graded as critically-low quality; 2.7% SRs were of low quality; and only 0.7% SRs were of high quality. Further improvement is needed in publishing a priori protocol of review, explaining the selection of study design, conducing a comprehensive literature search, documenting lists of excluded studies, conducting MA with appropriate statistical pooling methods, and reporting funding sources for included primary studies. This is because less than 10% of included SRs satisfied these domain-specific items. Cochrane reviews, SRs received funding support from Europe, SRs conducted by more authors, and SRs published in higher impact factor journals were positively associated with the overall methodological quality.

### Comparisons with other methodological survey on systematic review rigour

The proportion of CHM SRs with high or moderate overall methodological quality (0.7%) resembled appraisal results on acupuncture SRs (0.9%) [36]. Nevertheless, among AMSTAR 2 critical domains, SRs on CHM has a better performance than acupuncture on (i) using comprehensive literature search strategies (4.1% versus 3.8%); (ii) using appropriate methods for statistical combination of results (6.1% versus 5.7%); (iii) accounting for risk of bias among primary studies when interpreting synthesised results (78.4% versus 73.6%); and (iv) carrying out adequate investigations of publication bias and discussing its likely impact on SR results (60.1% versus 21.7%). As compared to a previous methodological survey on CHM SR using the original AMSTAR [18], recent CHM SRs performed no better than those published during 1993 to 2013, except in reporting potential sources of conflict of interest (97.3% versus 3.5%).

### Recommendations for future systematic reviews

#### Developing and registering a priori protocols with justifications for deviations

Development of a priori SR protocols allows review authors to document detailed methodology in advance, as well as minimising impact of review authors’ biases influenced by their existing knowledge in the field [37]. To increase transparency, reduce publication bias, and prevent unnecessary duplication [38], it is recommended that review authors should register SR protocols on PROSPERO [39], or publish the protocols in open access journals [40]. Unfortunately, only 8.8% included SRs provided a priori protocols together with justifications for deviations. Authors are also encouraged to specify rationale for selecting particular study designs in the protocols, as none of the included SRs fulfilled this AMSTAR 2 domain.

#### Conducing comprehensive literature search

Comprehensive literature searches enable review authors to obtain a comprehensive set of primary studies for answering a particular PICO question [37]. Primary studies reporting positive outcomes are more likely to be published irrespective of their methodological rigour [41]. Retrieving eligible unpublished studies through grey literature and reference lists searches, and consulting experts in the field are also critical in reducing publication bias [30, 37]. Nonetheless, only 4.1% included SRs performed literature search which was considered comprehensive by AMSTAR 2 standard.

It is also noteworthy that 70.3% SRs did not clarify publication language of included primary studies, which might give rise to language bias [37]. Previous studies demonstrated that SRs including only English primary studies may cause overestimation [42] or underrepresentation [43] of effect estimates. Inclusion of both English and non-English primary studies in SRs would increase the generalisability and applicability of the intervention effects [37]. This is particularly relevant for SRs of CHM since a lack of literature search among Chinese databases may cause a change in conclusion [44].

In our sample, only 16.2% included SRs reported a reproducible full Boolean search strategy which have been implemented. According to the recently updated PRISMA 2020 Statement, full search strategies for all databases, registers, and websites should be presented in SRs [45]. For any filters and limits applied to the search strategies, such as publication status or language of primary studies, justifications should also be reported based on the SRs’ eligibility criteria.

#### Providing a list of excluded studies with rationales

A list of excluded studies should be provided along with rationales to reduce subjectivity and ensure transparency of study selection in SRs [45]. This process may reduce selective omission of primary studies with unfavourable results [30, 37]. Other potential sources of bias or errors stemmed from inappropriate exclusion of relevant studies can also be traced based on the list [30, 37]. Unfortunately, our finding highlighted that such practice was only implemented by 1.4% included SRs. Future review authors should put more effort in addressing this limitation.

#### Conducting meta-analyses with appropriate statistical methods

MA is a statistical process which combines results of primary studies quantitatively within a SR. [37] Nevertheless, validity of the pooled results will be questionable if review authors choose an inappropriate statistical method to conduct MAs [46]. For instance, performing a fixed-effect, instead of random-effect, MA among primary studies conducted in different centres may undermine trustworthiness of effect estimates [37]. Our study illustrated that over 90% included SRs performed MAs using inappropriate pooling method. Future teams of review authors should be composed of both content experts and methods experts, such as clinical epidemiologists, information specialist, and statisticians [37, 47]. Recruiting these methodologists into research teams may improve methodological rigour of SRs.

#### Reporting sources of funding among included primary studies

Solid evidence demonstrated that RCTs sponsored by commercial sources are more likely to draw favourable conclusions benefiting the sponsors [48], especially for pharmacological interventions. A cross-sectional study of RCTs also indicated that financial ties between principal investigators and industry are independently associated with positive trial results. This might jeopardise the methodological rigour of studies, and possibly lead to biased conclusions [30]. It is important for SR authors to clarify and report such relationship, if any. Nevertheless, only 4.7% of our SR sample documented sources of funding among included primary studies. We recommend that future review authors should report funding transparently.

#### Adherence to international methodological and reporting standards

Overall speaking, the Cochrane Handbook for Systematic Reviews of Interventions should be used to guide the general conduct of SRs [37]. The PRISMA 2020 Statement should also be followed for ensuring transparent SR reporting [45].

### Strengths and limitations

This methodological survey has several strengths. Firstly, methodological quality of both Cochrane and non-Cochrane CHM SRs were assessed using the latest validated critical appraisal tool, AMSTAR 2. Secondly, the performance of included SRs on each AMSTAR 2 item was reported separately to inform improvement needs in specific aspects. Thirdly, SR-like methods, such as comprehensive search in representative electronic databases, duplicate eligibility assessment and data extraction, were implemented to minimise bias. Lastly, as an update a previous methodological survey [18], we have addressed the knowledge gap regarding the lack of overall methodological improvement among on CHM SR over the past few years.

This study has several limitations. Firstly, we did not include or appraise CHM SRs published before 2018. However, this may not be a critical issue since the Cochrane Collaboration policy recommends that clinical decision should be made based on SRs published within two years [37]. Secondly, our search was limited to international English databases without searching regional and subject-specific databases on CHM. This might thus reduce the generalisability of the results [37], but indeed the sample we assessed is clearly representative of SRs indexed in major international databases. Thirdly, we did not search the Allied and Complementary Medicine (AMED) Database. This might have led to sampling bias as certain eligible CHM SRs indexed only in this database [49]. Yet, we believe that the current sample represents the commonly utilised SRs by clinicians and policy makers internationally. Fourthly, without evaluating SRs without MA, this methodological survey might have under- or over-estimated the overall methodological quality of recent CHM SRs. In the future, SRs without MA can be appraised by an updated AMSTAR 2 which will incorporate requirements from the SWiM guideline. Lastly, AMSTAR 2 assessment was based exclusively on published information, and low reporting quality among included SRs might have hampered the accuracy of our appraisal results. That said, this limitation could have been minimised via seeking additional information from SR authors. Indeed, better compliance to the PRISMA reporting guideline among SR authors will facilitate more reliable assessment in the future.

### Implications for practice and research

Currently, development of evidence-based application of CHM has been encouraged by the Chinese government, as documented in *The Construction Plan for the Chinese Medicine Highlands in the Guangdong-Hong Kong-Macao Greater Bay Area (2020-2025)* [13] and *The Opinions of the Chinese Communist Party Central Committee and the State Council on Promoting the Preservation, Innovation, and Development of Traditional Chinese Medicine* [14]. While a solid evidence base supporting CHM use is key to these policy developments, our findings highlighted that methodological quality of recent SRs on CHM is low. It is likely that these methodological flaws have caused overestimation or underestimation of intervention effect, which may then mislead decision making [19]. Policy makers and healthcare professionals should beware of SRs quality before adopting the results in clinical practice [30].

To improve methodological quality of future RCTs and SRs on CHM, substantial professional resources and funding should be allocated for supporting research training in clinical epidemiology and evidence-based healthcare among Chinese medicine researchers. Apart from capacity building, journal editors and peer reviewers are strongly recommended to follow the updated methodological and reporting standards when assessing submissions [30, 37], such that quality of future publications would benefit from the peer reviewing process.

## Conclusions

The overall methodological quality of SRs on CHM published is far from satisfactory, with only 0.7% of SRs being assessed as high quality. Imminent improvements are needed to (i) develop and register a priori SR protocol with justifications for deviations and selection of study design, (ii) conduct comprehensive literature searches, (iii) provide lists of excluded studies with rationales, (iv) conduct MAs with appropriate statistical methods, and (v) report funding sources among included primary studies. To accomplish these, joint efforts from policy makers, review authors, journal editors, and peer reviewers are necessary.

## Supplementary Information

**Additional file 1: Table S1**. Search strategies on three electronic databases during January 2018 to March 2020. **Table S2**. Data extraction form of bibliographical characteristics. **Table S3**. List of included systematic reviews on Chinese herbal medicine. **Table S4**. Conditions and study arms of the included systematic reviews on Chinese herbal medicine. **Table S5**. List of excluded systematic reviews on Chinese herbal medicine after assessing full text for eligibility and reasons for exclusion.

**Additional file 2.**

## Data Availability

All data generated or analysed during this study are included in this published article and its supplementary information files.

## Abbreviations

AMSTAR 2: Assessing the Methodological Quality of Systematic Reviews 2
CM: Chinese medicine
CHM: Chinese herbal medicine
MA: Meta-analysis
PICO: Population, intervention, comparison, outcome
RCT: Randomised controlled trials
SR: Systematic review
TCIM: Traditional, complementary, and integrative medicine
WHO: World Health Organization
