# Supplementary material for: Methodological quality of systematic reviews on Chinese herbal medicine: a methodological survey
Source: BMC Complement Med Ther. 2022 Feb 23;22:48. doi: 10.1186/s12906-022-03529-w (PMC8867833; doi:10.1186/s12906-022-03529-w)
Supplement: Supplementary file 1 — Additional file 1: Table S1. Search strategies on three electronic databases during January 2018 to March 2020. Table S2. Data extraction form of bibliographical characteristics. Table S3. List of included systematic reviews on Chinese herbal medicine. Table S4. Conditions and study arms of the included systematic reviews on Chinese herbal medicine. Table S5. List of excluded systematic reviews on Chinese herbal medicine after assessing full text for eligibility and reasons for exclusion. [file 12906_2022_3529_MOESM1_ESM.docx]

**Supplementary file 1**

**Table S1.** Search strategies on three electronic databases during January 2018 to March 2020

**Table S2.** Data extraction form of bibliographical characteristics

**Table S3.** List of included systematic reviews on Chinese herbal medicine

**Table S4.** Conditions and study arms of the included systematic reviews on Chinese herbal medicine

**Table S5.** List of excluded systematic reviews on Chinese herbal medicine after assessing full text for eligibility and reasons for exclusion

**Table S1.** Search strategies on three electronic databases during January 2018 to March 2020

(1) Cochrane Database of Systematic Reviews:

“Chinese herb*” or “herb*” or “Traditional Chinese medic*” or “phytother*” or “(chinese adj5 (traditional or medic*))” or “(plant or plants)” or “(traditional adj5 medic*)” or “Chinese medic*” or “oriental medic*” or “herbaceous agent” or “medicinal plant*” with Cochrane Library publication date in The last 2 years, in Cochrane Reviews yielded 424 citations.

(2) MEDLINE:

| **#** | **Search Statement** | **Results** |
| --- | --- | --- |
| 1 | MEDLINE.tw. | 94422 |
| 2 | systematic review.tw. | 114409 |
| 3 | meta analysis.pt. | 112011 |
| 4 | 1 or 2 or 3 | 224528 |
| 5 | exp Drugs, Chinese Herbal/ | 42557 |
| 6 | Chinese herb*.mp. | 45758 |
| 7 | exp Medicine, Chinese Traditional/ | 18511 |
| 8 | Traditional Chinese medic*.mp. | 17495 |
| 9 | exp Phytotherapy/ | 38958 |
| 10 | phytother*.mp. | 39092 |
| 11 | (chinese adj5 (traditional or medic*)).mp. | 40996 |
| 12 | (herbs or herbal).mp. | 69396 |
| 13 | (plant or plants).mp. | 706891 |
| 14 | (traditional adj5 medic*).mp. | 58689 |
| 15 | 5 or 6 or 7 or 8 or 9 or 10 or 11 or 12 or 13 or 14 | 787019 |
| 16 | 4 and 15 | 4794 |
| 17 | limit 16 to (humans and yr="2018 -Current") | 987 |

(3) EMBASE:

| **#** | **Search Statement** | **Results** |
| --- | --- | --- |
| 1 | meta-analysis.tw. | 186158 |
| 2 | systematic review.tw. | 184304 |
| 3 | 1 or 2 | 296428 |
| 4 | exp Chinese medicine/ | 38189 |
| 5 | exp oriental medicine/ | 2613 |
| 6 | exp herbaceous agent/ | 50560 |
| 7 | exp medicinal plant/ | 233829 |
| 8 | exp Chinese herb/ | 4767 |
| 9 | Chinese medic*.mp. | 63683 |
| 10 | oriental medic*.mp. | 3943 |
| 11 | herbaceous agent.mp. | 50562 |
| 12 | medicinal plant*.mp. | 94507 |
| 13 | Chinese herb*.mp. | 16186 |
| 14 | herb*.mp. | 183467 |
| 15 | 4 or 5 or 6 or 7 or 8 or 9 or 10 or 11 or 12 or 13 or 14 | 409748 |
| 16 | 3 and 15 | 4361 |
| 17 | limit 16 to (human and yr="2018 -Current") | 1162 |

**Table S2.** Data extraction form of bibliographical characteristics

| **Bibliographical features** | **Answers** |
| --- | --- |
| 1. Is it a Cochrane systematic review? | ☐ Yes / ☐ No |
| 1. Is it an update of previous review? | ☐ Yes / ☐ No |
| 1. Year of systematic review’s publication |  |
| 1. Impact factor of systematic review’s journal in the year before its publication |  |
| 1. The number of systematic review’s authors |  |
| 1. Location of corresponding author | ☐ Europe  ☐ America  ☐ Asia  ☐ Oceania  ☐ Africa  ☐ Not reported |
| 1. The number of included primary studies |  |
| 1. The number of participants in included primary studies |  |
| 1. Has harm been considered in the systematic review? | ☐ Yes / ☐ No |
| 1. Result of the first primary outcome of the systematic review | ☐ No significant difference between CHM intervention and control  ☐ In favour of CHM intervention  ☐ In favour of CHM intervention with reservation |
| 1. Funding location of the systematic review | ☐ Europe  ☐ America  ☐ Asia  ☐ Oceania  ☐ Africa  ☐ Not reported |
| 1. The nature of financial support for systematic review | ☐ Not reported  ☐ Not-for-proﬁt source  ☐ No funding  ☐ For-proﬁt source |
| 1. Did the systematic review’s literature search involve English databases? | ☐ Yes / ☐ No |
| 1. Did the systematic review’s literature search involve non-English databases? | ☐ Yes / ☐ No |
| 1. Report of coverage year of the search strategies | ☐ Yes (starting and ending years)  ☐ Partially (only starting years)  ☐ Not mentioned |
| 1. Report of search terms | ☐ No research term  ☐ Topics / free text / keywords / MeSH  ☐ Full Boolean  ☐ Readers are referred elsewhere for full search strategy |
| 1. Eligibility criteria based on language of publication | ☐ English only  ☐ Language other than English  ☐ English and other languages  ☐ Not reported |
| 1. Risk of bias tool for primary studies assessment | ☐ Cochrane risk of bias  ☐ Jaded scale  ☐ Schulz approach  ☐ Effective Public Health Practice Project Quality Assessment Tool  ☐ Juni  ☐ Chalmer scale  ☐ Pedro Scale  ☐ Delphi list  ☐ More than one tools  ☐ Others |
| 1. Is there any PRISMA-like flow diagram? | ☐ Yes / ☐ No |

*Keys:* RCT = randomised controlled trial; MeSH = National Library of Medical Subject Headings; PRISMA= Preferred Reporting Items for Systematic Reviews and Meta-analysis.

**Table S3.** List of included systematic reviews on Chinese herbal medicine

| **ID** | **Name of systematic reviews** | **Years** | **Publication Journal** | **Source of funding** | **Journal impact factor** | **Number of primary studies** | **Number of patients** |
| --- | --- | --- | --- | --- | --- | --- | --- |
|  |  |  |  |  |  |  |  |
| 1 | Efficacy and safety of compound Kushen injection combined with chemotherapy on postoperative Patients with breast cancer: A meta-analysis of randomized controlled trials | 2019 | Medicine | Not-for-profit | 1.87 | 16 | 1315 |
| 2 | Zao Ren An Shen for insomnia: a systematic review with meta-analysis | 2020 | Sleep Medicine | No funding | 3.04 | 19 | 1780 |
| 3 | Add-on effects of Chinese herbal medicine for post-stroke spasticity: A systematic review and meta-analysis | 2019 | Frontiers in Pharmacology | Not-for-profit | 3.85 | 35 | 2457 |
| 4 | Evidence of Astragalus injection combined platinum-based chemotherapy in advanced non-small cell lung cancer patients: A systematic review and meta-analysis | 2019 | Medicine | Not-for-profit | 1.87 | 19 | 1635 |
| 5 | Evaluation of the adjunctive effect of Xing Nao Jing Injection for viral encephalitis: A systematic review and meta-analysis of randomized controlled trials | 2019 | Medicine | Not-for-profit | 1.87 | 23 | 1757 |
| 6 | Traditional Chinese herbal medicine for vascular dementia | 2018 | Cochrane Database of Systematic Reviews | Not-for-profit | 6.75 | 47 | 3581 |
| 7 | Qishenyiqi dripping pill improves ventricular remodeling and function in patients with chronic heart failure: A pooled analysis | 2019 | Medicine | Not-for-profit | 1.87 | 12 | 877 |
| 8 | Does the Oral Administration of Ginger Reduce Chemotherapy-Induced Nausea and Vomiting?: A Meta-analysis of 10 Randomized Controlled Trials | 2019 | Cancer Nursing | Not reported | 2.02 | 10 | 1313 |
| 9 | Efficacy and safety of Zaoren Anshen capsules in the treatment of insomnia: A meta-analysis of randomized controlled trials | 2020 | Medicine | Not-for-profit | 1.55 | 13 | 1175 |
| 10 | Effects of Shenfu injection on chemotherapy-induced adverse effects and quality of life in patients with advanced non-small cell lung cancer: A systematic review and meta-analysis | 2018 | Journal of Cancer Research and Therapeutics | No funding | 0.84 | 16 | 948 |
| 11 | Efficacy and safety of Xuebijing injection combined with ulinastatin as adjunctive therapy on sepsis: A systematic review and meta-analysis | 2018 | Frontiers in Pharmacology | Not reported | 3.83 | 17 | 1247 |
| 12 | Compound Kushen injection combined with platinum-based chemotherapy for stage III/IV non-small cell lung cancer: A meta-analysis of 37 RCTs following the PRISMA guidelines | 2020 | Journal of Cancer | Not-for-profit | 3.57 | 37 | 3272 |
| 13 | Traditional Chinese medicine yimucao injection combined with western medicine for preventing postpartum hemorrhage after cesarean section: A systematic review and meta-analysis | 2019 | Evidence-based Complementary and Alternative Medicine | Not reported | 1.98 | 48 | 7330 |
| 14 | Chinese herbal medicine for myasthenia gravis: A systematic review and meta-analysis | 2018 | Frontiers in Pharmacology | Not-for-profit | 3.83 | 14 | 1039 |
| 15 | Systematic review and meta-analysis of the efficacy and safety of biqi capsule in rheumatoid arthritis patients | 2018 | Experimental and Therapeutic Medicine | Not-for-profit | 1.41 | 5 | 552 |
| 16 | Chinese herbal medicine for epidermal growth factor receptor inhibitor-induced skin rash in patients with malignancy: An updated meta-analysis of 23 randomized controlled trials | 2019 | Complementary Therapies in Medicine | Not-for-profit | 1.98 | 23 | 1392 |
| 17 | Effect of Chinese medicine injection for preventing contrast-induced nephropathy after coronary angiography/percutaneous coronary intervention: A systematic review and meta-analysis | 2018 | International Journal of Clinical and Experimental Medicine | Not-for-profit | 0 | 13 | 2022 |
| 18 | Efficacy of ginger (zingiber officinale) in ameliorating chemotherapy-induced nausea and vomiting and chemotherapy-related outcomes: a systematic literature review update and meta-analysis | 2018 | Integrative Cancer Therapies | Not-for-profit | 2.66 | 18 | 1652 |
| 19 | Efficacy and safety of Modified Tongxie Yaofang in diarrhea-predominant irritable bowel syndrome management: A meta-analysis of randomized, positive medicine-controlled trials | 2018 | PLoS one | Not-for-profit | 2.77 | 23 | 1972 |
| 20 | Shexiang Baoxin Pills as an Adjuvant Treatment for Chronic Heart Failure: A System Review and Meta-Analysis | 2018 | Evidence-Based Complementary and Alternative Medicine | Not-for-profit | 2.06 | 27 | 2637 |
| 21 | Effects of Shengmai injection add-on therapy to chemotherapy in patients with non-small cell lung cancer: a meta-analysis | 2018 | Supportive Care in Cancer | Not-for-profit | 2.68 | 15 | 898 |
| 22 | Efficacy of herbal medicine (Gegen Qinlian Decoction) on ulcerative colitis: A systematic review of randomized controlled trials | 2019 | Medicine | Not-for-profit | 1.87 | 22 | 2028 |
| 23 | A meta-analysis of the clinical efficacy of TCM decoctions made from formulas in the liuwei dihuang wan categorized formulas in treating diabetic nephropathy proteinuria | 2018 | Evidence-based Complementary and Alternative Medicine | Not-for-profit | 2.06 | 14 | 918 |
| 24 | Herbal Formula Modified Buzhong-Yiqi-Tang for Functional Constipation in Adults: A Meta-Analysis of Randomized Controlled Trials | 2018 | Evidence-based Complementary and Alternative Medicine | Not-for-profit | 2.06 | 25 | 2089 |
| 25 | Safety and Clinical Efficacy of Yangxue Qingnao Granules in the Treatment of Chronic Cerebral Circulation Insufficiency: A Systematic Review and Meta-Analysis | 2019 | Evidence-based Complementary and Alternative Medicine | Not-for-profit | 1.98 | 15 | 1211 |
| 26 | Xi huang pills enhance the tumor treatment efficacy when combined with chemotherapy: A meta-analysis and systematic review | 2018 | Journal of Cancer Research and Therapeutics | Not-for-profit | 0.84 | 15 | 815 |
| 27 | Can External Use of Chinese Herbal Medicine Prevent Cumulative Peripheral Neuropathy Induced by Oxaliplatin? A Systematic Literature Review With Meta-analysis | 2019 | Integrative Cancer Therapies | Not-for-profit | 2.63 | 9 | 700 |
| 28 | Does ginger supplementation lower blood pressure? A systematic review and meta-analysis of clinical trials | 2019 | Phytotherapy Research | No funding | 3.77 | 10 | 345 |
| 29 | Qingkailing Injection for Treatment | 2018 | Chinese Journal of Integrative Medicine | Not-for-profit | 1.35 | 7 | 992 |
| 30 | Goshajinkigan for reducing chemotherapy-induced peripheral neuropathy: a systematic review and meta-analysis | 2018 | International Journal of Clinical Oncology | Not-for-profit | 2.61 | 5 | 386 |
| 31 | Rhubarb combined with trypsin inhibitor for severe acute pancreatitis: A systematic review and meta-analysis | 2018 | Phytotherapy Research | No funding | 3.35 | 15 | 912 |
| 32 | Effect of ginger in the treatment of nausea and vomiting compared with vitamin B6 and placebo during pregnancy: a meta-analysis | 2020 | Journal of Maternal-Fetal & Neonatal Medicine | Not-for-profit | 1.74 | 13 | 1174 |
| 33 | Dietary ginger as a traditional therapy for blood sugar control in patients with type 2 diabetes mellitus: A systematic review and meta-analysis | 2019 | Medicine | No funding | 1.87 | 8 | 454 |
| 34 | Efficacy and safety of Shenfu injection for septic shock: A systematic review and meta-analysis of randomized controlled trials | 2019 | American Journal of Emergency Medicine | Not-for-profit | 1.65 | 19 | 1505 |
| 35 | Xuebijing injection for acute organophosphorus pesticide poisoning: A systematic review and meta-Analysis | 2019 | Annals of Translational Medicine | Not-for-profit | 3.69 | 26 | 1880 |
| 36 | Systematic evaluation of therapeutic efficacy and safety of traditional Chinese medicine injection (TCMI) combined with oxaliplatin-containing chemotherapy in the treatment of colorectal cancer | 2018 | International Journal of Clinical and Experimental Medicine | Not-for-profit | 0 | 77 | 6014 |
| 37 | Shengmai injection as an adjunctive therapy for the treatment of chronic obstructive pulmonary disease: A systematic review and meta-analysis | 2019 | Complementary Therapies in Medicine | Not-for-profit | 1.98 | 23 | 1804 |
| 38 | Efficacy and safety of Sijunzi decoction for peptic ulcers: A systematic review and meta-analysis | 2018 | Journal of Traditional Chinese Medical Sciences | Not-for-profit | 0 | 14 | 1476 |
| 39 | Kanglaite injection plus platinum-based chemotherapy for stage III/IV non-small cell lung cancer: A meta-analysis of 27 RCTs | 2020 | Phytomedicine | Not-for-profit | 4.27 | 27 | 2243 |
| 40 | The effects of ginger supplementation on markers of inflammatory and oxidative stress: A systematic review and meta-analysis of clinical trials | 2020 | Phytotherapy Research | Not reported | 4.09 | 20 | 888 |
| 41 | Does Adjuvant Treatment with Chinese Herbal Medicine to Antidiabetic Agents Have Additional Benefits in Patients with Type 2 Diabetes? A System Review and Meta-Analysis of Randomized Controlled Trials | 2019 | Evidence-based Complementary and Alternative Medicine | Not-for-profit | 1.98 | 10 | 2004 |
| 42 | Efficacy and Safety of Xiaoyao Formula as an Adjuvant Treatment for Post-Stroke Depression: A Meta-Analysis | 2018 | Explore | Not-for-profit | 0.99 | 7 | 607 |
| 43 | Danggui-Shaoyao-San for dementia: A PRISMA-compliant systematic review and meta-analysis | 2020 | Medicine | Not-for-profit | 1.55 | 9 | 567 |
| 44 | Xiao Chai Hu Tang, a herbal medicine, for chronic hepatitis B | 2019 | Cochrane Database of Systematic Reviews | Not-for-profit | 7.76 | 10 | 934 |
| 45 | Efficacy and safety of Sihogayonggolmoryeo-tang (Saikokaryukotsuboreito, Chai-Hu-Jia-Long-Gu-Mu-Li-Tang) for post-stroke depression: A systematic review and meta-analysis | 2019 | Scientific Reports | Not-for-profit | 4.01 | 21 | 1644 |
| 46 | Herbal medicine (Gegen-decoction) for treating cervical spondylosis: A systematic review and meta-analysis of randomized controlled trials | 2018 | European Journal of Integrative Medicine | Not-for-profit | 0.70 | 5 | 454 |
| 47 | Herbal medicine (Hyeolbuchukeo-tang or Xuefu Zhuyu decoction) for treating primary dysmenorrhea: A systematic review and meta-analysis of randomized controlled trials | 2019 | Medicine | Not-for-profit | 1.87 | 8 | 1048 |
| 48 | Efficacy and Safety of Chinese Herbs for the Prevention of the Risk of Renal Damage in Henoch-Schonlein Purpura in Children: Meta-Analysis of Randomized Controlled Trials and GRADE Evaluation | 2019 | Evidence-based Complementary and Alternative Medicine | Not reported | 1.98 | 39 | 3643 |
| 49 | Efficacy and safety of Xuebijing injection (a Chinese patent) for sepsis: A meta-analysis of randomized controlled trials | 2018 | Journal of Ethnopharmacology | Not-for-profit | 3.12 | 16 | 1144 |
| 50 | Efficacy and safety of TCM Yangxin Anshen Therapy for insomnia: A systematic review and meta-analysis | 2020 | Medicine | Not-for-profit | 1.55 | 14 | 1549 |
| 51 | Gardenia fructus antidepressant formula for depression in diabetes patients: A systematic review and meta-analysis | 2020 | Complementary Therapies in Medicine | Not-for-profit | 2.06 | 12 | 822 |
| 52 | Twelve Chinese herbal preparations for the treatment of depression or depressive symptoms in cancer patients: a systematic review and meta-analysis of randomized controlled trials | 2019 | BMC Complementary and Alternative Medicine | Not-for-profit | 2.48 | 18 | 1441 |
| 53 | Chinese herbal formulae for the treatment of menopausal hot flushes: A systematic review and meta-analysis | 2019 | PLoS one | No funding | 2.78 | 19 | 2469 |
| 54 | Potential effectiveness of Chinese patent medicine Tongxinluo capsule for secondary prevention after acute myocardial infarction: A systematic review and meta-analysis of randomized controlled trials | 2018 | Frontiers in Pharmacology | Not-for-profit | 3.83 | 19 | 1877 |
| 55 | Xiyanping plus Azithromycin Chemotherapy in Pediatric Patients with Mycoplasma pneumoniae Pneumonia: A Systematic Review and Meta-Analysis of Efficacy and Safety | 2019 | Evidence-based Complementary and Alternative Medicine | Not-for-profit | 1.98 | 9 | 963 |
| 56 | Effectiveness of traditional Chinese medicine Jinlida granules as an add-on therapy for type 2 diabetes: A system review and meta-analysis of randomized controlled trials | 2019 | Journal of Diabetes | Not-for-profit | 3.30 | 15 | 1810 |
| 57 | Huangqi Guizhi Wuwu Decoction for treating cervical radiculopathy: A systematic review and meta-analysis of randomized controlled trials | 2020 | Medicine | Not-for-profit | 1.55 | 8 | 783 |
| 58 | Angong Niuhuang Pill as adjuvant therapy for treating acute cerebral infarction and intracerebral hemorrhage: A meta-analysis of randomized controlled trials | 2019 | Journal of Ethnopharmacology | Not reported | 3.41 | 18 | 1601 |
| 59 | Efficacy and safety of Kanglaite injection combined with radiochemotherapy in the treatment of advanced pancreatic cancer: A PRISMA-compliant meta-analysis | 2019 | Medicine | No funding | 1.87 | 16 | 960 |
| 60 | Effects of Aidi Injection with Western Medical Therapies on Quality of Life for Patients with Primary Liver Cancer: A Systematic Review and Meta-Analysis | 2019 | Chinese Journal of Integrative Medicine | Not-for-profit | 1.45 | 24 | 1812 |
| 61 | Traditional Chinese medicine for Bradyarrhythmia: Evidence and potential mechanisms | 2018 | Frontiers in Pharmacology | Not-for-profit | 3.83 | 121 | 11138 |
| 62 | Shenqi Fuzheng Injection in the Treatment of Breast Cancer: A Meta-analysis of Randomized Controlled Trials | 2019 | Integrative Cancer Therapies | Not-for-profit | 2.63 | 31 | 2543 |
| 63 | Clinical Efficacy of Jinshuibao Capsules Combined with Angiotensin Receptor Blockers in Patients with Early Diabetic Nephropathy: A Meta-Analysis of Randomized Controlled Trials | 2018 | Evidence-based Complementary and Alternative Medicine | Not-for-profit | 2.06 | 26 | 2198 |
| 64 | Sanjin tablet combined with antibiotics for treating patients with acute lower urinary tract infections: A meta-analysis and GRADE evidence profile | 2020 | Experimental and Therapeutic Medicine | Not-for-profit | 1.79 | 8 | 790 |
| 65 | Tongxinluo Capsule for Cardiac Syndrome X: A Systematic Review and Meta-Analysis | 2018 | Chinese Journal of Integrative Medicine | Not-for-profit | 1.35 | 12 | 696 |
| 66 | Oral Chinese Herbal Medicine for Heart Failure with Preserved Ejection Fraction: A Meta-Analysis | 2019 | Chinese Journal of Integrative Medicine | Not-for-profit | 1.45 | 16 | 1320 |
| 67 | Efficacy and safety of motherwort injection add-on therapy to carboprost tromethamine for prevention of post-partum blood loss: A meta-analysis of randomized controlled trials | 2019 | Journal of Obstetrics and Gynaecology Research | Not reported | 1.12 | 8 | 1276 |
| 68 | Add-on effect of Guizhi Fuling formula to mifepristone for endometriosis: A meta-analysis of randomized controlled trials | 2019 | Medicine | No funding | 1.87 | 10 | 1052 |
| 69 | Effects of ginseng supplementation on selected markers of inflammation: A systematic review and meta-analysis | 2019 | Phytotherapy Research | No funding | 3.77 | 8 | 409 |
| 70 | A systematic review on Chinese herbal treatment for radiotherapy-induced xerostomia in head and neck cancer patients | 2018 | Complementary Therapies in Clinical Practice | No funding | 1.70 | 15 | 1054 |
| 71 | Traditional Chinese Patent Medicine for Treating Impaired Glucose Tolerance: A Systematic Review and Meta-Analysis of Randomized Controlled Trials | 2018 | Journal of Alternative and Complementary Medicine | Not-for-profit | 1.50 | 18 | 3172 |
| 72 | Clinical evidence on the effects of saffron (Crocus sativus L.) on cardiovascular risk factors: A systematic review meta-analysis | 2019 | Pharmacological Research | No funding | 5.57 | 11 | 622 |
| 73 | The effect of ginger supplementation on lipid profile: A systematic review and meta-analysis of clinical trials | 2018 | Phytomedicine | No funding | 3.61 | 14 | 898 |
| 74 | KangFuXin Liquid in the Treatment of Diabetic Foot Ulcer: A Systematic Review and Meta-Analysis | 2019 | Evidence-based Complementary and Alternative Medicine | Not-for-profit | 1.98 | 11 | 889 |
| 75 | Effects of saffron (Crocus sativus) on sexual dysfunction among men and women: A systematic review and meta-analysis | 2019 | Avicenna Journal of Phytomedicine | No funding | 0 | 5 | 173 |
| 76 | Efficacy of Suxiao Jiuxin Pill on Coronary Heart Disease: A Meta-Analysis of Randomized Controlled Trials | 2018 | Evidence-based Complementary and Alternative Medicine | No funding | 2.06 | 41 | 6276 |
| 77 | Chinese herbal medicines for the treatment of non-structural abnormal uterine bleeding in perimenopause: A systematic review and a meta-analysis | 2018 | Complementary Therapies in Medicine | Not-for-profit | 2.08 | 15 | 1344 |
| 78 | Chinese herbal medicines of supplementing Qi and nourishing Yin combined with chemotherapy for non-small cell lung cancer: A meta-analysis and systematic review | 2019 | Journal of Cellular Biochemistry | Not-for-profit | 3.45 | 41 | 2607 |
| 79 | Corn silk tea for hypertension: A systematic review and meta-analysis of randomized controlled trials | 2019 | Evidence-based Complementary and Alternative Medicine | Not reported | 1.98 | 5 | 567 |
| 80 | Corn silk decoction for blood lipid in patients with angina pectoris: A systematic review and meta-analysis | 2019 | Phytotherapy Research | Not reported | 3.77 | 4 | 800 |
| 81 | Chinese herbal medicine for headache: A systematic review and meta-analysis of high-quality randomized controlled trials | 2019 | Phytomedicine | Not-for-profit | 4.18 | 30 | 3447 |
| 82 | Treatment of depression with Chai Hu Shu Gan San: a systematic review and meta-analysis of 42 randomized controlled trials | 2018 | BMC Complementary and Alternative Medicine | No funding | 2.11 | 42 | 3234 |
| 83 | Effectiveness and safety of Chinese herbal medicine for pediatric adenoid hypertrophy: A meta-analysis | 2019 | International Journal of Pediatric Otorhinolaryngology | No funding | 1.23 | 13 | 1083 |
| 84 | Randomized Controlled Trials of Tianma Gouteng Decoction Combined with Nifedipine in the Treatment of Primary Hypertension: A Systematic Review and Meta-Analysis | 2020 | Evidence-based Complementary and Alternative Medicine | Not-for-profit | 1.81 | 14 | 1537 |
| 85 | Traditional Chinese medicine injections in the treatment of diabetic foot: A systematic review and meta-analysis | 2018 | Evidence-based Complementary and Alternative Medicine | Not-for-profit | 2.06 | 17 | 1294 |
| 86 | Efficacy and Safety Evaluation of Taohong Siwu Decoction for Patients with Angina Pectoris: A Meta-Analysis of Randomized Controlled Trials | 2019 | Chinese Journal of Integrative Medicine | Not-for-profit | 1.45 | 12 | 959 |
| 87 | The Efficacy of Saffron in the Treatment of Mild to Moderate Depression: A Meta-analysis | 2019 | Planta Medica | Not-for-profit | 2.75 | 11 | 531 |
| 88 | The Effects of a Fixed Combination of Berberis aristata and Silybum marianum on Dyslipidaemia - A Meta-analysis and Systematic Review | 2020 | Planta Medica | Not-for-profit | 2.69 | 4 | 491 |
| 89 | Efficacy and safety of Chinese herbal medicine for primary intracerebral hemorrhage: A systematic review of randomized controlled trials | 2019 | Frontiers in Pharmacology | Not-for-profit | 3.85 | 45 | 4517 |
| 90 | Aidi injection plus platinum-based chemotherapy for stage IIIB/IV non-small cell lung cancer: A meta-analysis of 42 RCTs following the PRISMA guidelines | 2018 | Journal of Ethnopharmacology | Not-for-profit | 3.12 | 42 | 4081 |
| 91 | Adjuvant Therapy of Oral Chinese Herbal Medicine for Menopausal Depression: A Systematic Review and Meta-Analysis | 2018 | Evidence-based Complementary and Alternative Medicine | Not-for-profit | 2.06 | 22 | 1770 |
| 92 | Efficacy and safety of Chinese herbal medicine on ovarian cancer after reduction surgery and adjuvant chemotherapy: A systematic review and meta-analysis | 2019 | Frontiers in Oncology | Not-for-profit | 4.14 | 18 | 975 |
| 93 | The efficacy of Xue Fu Zhu Yu prescription for hyperlipidemia: A meta-analysis of randomized controlled trials | 2019 | Complementary Therapies in Medicine | Not-for-profit | 1.98 | 12 | 1305 |
| 94 | Danshen Formulae for Cancer: A Systematic Review and Meta-Analysis of High-Quality Randomized Controlled Trials | 2019 | Evidence-based Complementary and Alternative Medicine | Not-for-profit | 1.98 | 13 | 1045 |
| 95 | The effects of Chinese herbal medicines for treating diabetic foot ulcers: A systematic review of 49 randomized controlled trials | 2019 | Complementary Therapies in Medicine | Not-for-profit | 1.98 | 49 | 3646 |
| 96 | Erxian decoction, a Chinese herbal formula, for menopausal syndrome: An updated systematic review | 2019 | Journal of Ethnopharmacology | Not-for-profit | 3.41 | 16 | 1594 |
| 97 | Efficacy and safety of Chinese herbal medicine for depression: A systematic review and meta-analysis of randomized controlled trials | 2019 | Journal of Psychiatric Research | Not-for-profit | 3.92 | 40 | 3549 |
| 98 | Chinese herbal medicine combined with tadalafil for erectile dysfunction: a systematic review and meta-analysis | 2020 | Andrology | Not reported | 2.86 | 11 | 903 |
| 99 | Acupoint herbal patching during Sanfu Days on reducing frequency of acute asthma attack in children: A systematic review and meta-analysis | 2020 | Medicine | Not-for-profit | 1.55 | 11 | 882 |
| 100 | Kanglaite Injection Combined with Chemotherapy versus Chemotherapy Alone for the Improvement of Clinical Efficacy and Immune Function in Patients with Advanced Non-Small-Cell Lung Cancer: A Systematic Review and Meta-Analysis | 2020 | Evidence-based Complementary and Alternative Medicine | Not-for-profit | 1.81 | 25 | 2151 |
| 101 | Effect of Zhizhu Kuanzhong Capsules on Treatment of Functional Dyspepsia: A Meta-Analysis of Randomized Controlled Trials | 2019 | Chinese Journal of Integrative Medicine | Not reported | 1.45 | 23 | 2496 |
| 102 | Chinese Herbal Medicines for Restenosis After Percutaneous Coronary Intervention: A Meta-Analysis of Randomized Controlled Trials | 2019 | Journal of Alternative and Complementary Medicine | Not-for-profit | 1.87 | 11 | 1383 |
| 103 | Efficacy and Safety of Xiao Ai Ping Injection Combined with Chemotherapy in Advanced Gastric Cancer: A Systematic Review and Meta-Analysis | 2019 | Evidence-based Complementary and Alternative Medicine | Not-for-profit | 1.98 | 14 | 1097 |
| 104 | Yinzhihuang oral liquid combined with phototherapy for neonatal jaundice: a systematic review and meta-analysis of randomized clinical trials | 2018 | BMC Complementary and Alternative Medicine | Not-for-profit | 2.11 | 17 | 2561 |
| 105 | Meta analysis of clinical efficacy of traditional Chinese medicine in the treatment of aplastic anemia | 2018 | World Journal of Traditional Chinese Medicine | Not-for-profit | 0 | 2 | 100 |
| 106 | Curative efficacy and safety of traditional Chinese medicine xuebijing injections combined with ulinastatin for treating sepsis in the Chinese population: A meta-analysis | 2018 | Medicine | Not reported | 2.03 | 16 | 1335 |
| 107 | Clinical efficacy and safety of aidi injection combination with vinorelbine and cisplatin for advanced non-small-cell lung carcinoma: A systematic review and meta-analysis of 54 randomized controlled trials | 2020 | Pharmacological Research | Not-for-profit | 5.89 | 54 | 4053 |
| 108 | Clinical efficacy and safety of Aidi injection plus paclitaxel-based chemotherapy for advanced non-small cell lung cancer: A meta-analysis of 31 randomized controlled trials following the PRISMA guidelines | 2019 | Journal of Ethnopharmacology | Not-for-profit | 3.41 | 31 | 2058 |
| 109 | Can Aidi injection improve overall survival in patients with non-small cell lung cancer? A systematic review and meta-analysis of 25 randomized controlled trials | 2018 | Complementary Therapies in Medicine | Not-for-profit | 2.08 | 25 | 2662 |
| 110 | Yizhi decoction as a therapy for vascular dementia: A meta-analysis | 2018 | Tropical Journal of Pharmaceutical Research | Not reported | 0.44 | 14 | 1045 |
| 111 | Clinical Efficacy and Safety of Tanreqing Injection for Pulmonary Infection in Patients with Tuberculosis: A Meta-Analysis | 2018 | Journal of Alternative and Complementary Medicine | Not-for-profit | 1.50 | 26 | 2404 |
| 112 | Efficacy and safety of Chinese herbal medicine Xiao Yao San in hypertension: A systematic review and meta-analysis | 2019 | Phytomedicine | Not-for-profit | 4.18 | 17 | 1460 |
| 113 | Efficacy and safety of Chinese herbal medicine for patients with postmenopausal hypertension: A systematic review and meta-analysis | 2019 | Pharmacological Research | Not-for-profit | 5.57 | 39 | 3823 |
| 114 | Therapeutic efficacy and safety of traditional Chinese medicine classic herbal formula Longdanxiegan decoction for hypertension: A systematic review and meta-analysis | 2018 | Frontiers in Pharmacology | Not-for-profit | 3.83 | 9 | 863 |
| 115 | Efficacy and safety of Chinese patent medicine (Jinlong capsule) in the treatment of advanced hepatocellular carcinoma: a meta-analysis | 2020 | Bioscience Reports | Not-for-profit | 2.94 | 29 | 2488 |
| 116 | Chinese herbal medicine for Wilson's disease: A systematic review and meta-analysis | 2019 | Frontiers in Pharmacology | Not-for-profit | 3.85 | 18 | 1220 |
| 117 | Role of Xingnaojing combined with naloxone in treating intracerebral haemorrhage: A systematic review and meta-analysis of randomized controlled trials | 2018 | Medicine | Not-for-profit | 2.03 | 32 | 3068 |
| 118 | Chinese herbal bath therapy for the treatment of uremic pruritus: meta-analysis of randomized controlled trials | 2019 | BMC Complementary and Alternative Medicine | No funding | 2.48 | 17 | 970 |
| 119 | Oral Chinese Herbal Medicine for Depressive Disorder in Patients after Percutaneous Coronary Intervention: A Systematic Review and Meta-Analysis | 2019 | Chinese Journal of Integrative Medicine | Not-for-profit | 1.45 | 16 | 1443 |
| 120 | Chinese medicinal herbs in the treatment of diabetic cognitive impairment: A systematic review and meta-analysis | 2018 | Evidence-based Complementary and Alternative Medicine | Not reported | 2.06 | 9 | 576 |
| 121 | Acupuncture plus Chinese Herbal Medicine for Irritable Bowel Syndrome with Diarrhea: A Systematic Review and Meta-Analysis | 2019 | Evidence-based Complementary and Alternative Medicine | Not-for-profit | 1.98 | 21 | 1834 |
| 122 | Chinese herbal medicine for diabetic kidney disease: A systematic review and meta-analysis of randomised placebo-controlled trials | 2019 | BMJ Open | Not-for-profit | 2.38 | 20 | 2719 |
| 123 | Managing Depression with Bupleurum chinense Herbal Formula: A Systematic Review and Meta-Analysis of Randomized Controlled Trials | 2020 | Journal of Alternative and Complementary Medicine | Not-for-profit | 2.26 | 55 | 5572 |
| 124 | Efficacy and Safety of Fuzi Formulae on the Treatment of Heart Failure as Complementary Therapy: A Systematic Review and Meta-Analysis of High-Quality Randomized Controlled Trials | 2019 | Evidence-based Complementary and Alternative Medicine | Not-for-profit | 1.98 | 12 | 1490 |
| 125 | External application of Ruyi Jinhuang powder for phlebitis: A systematic review and meta-analysis | 2019 | Tropical Journal of Pharmaceutical Research | Not reported | 0.44 | 53 | 4119 |
| 126 | The effect of Chinese herbal medicine on quality of life and exercise tolerance in heart failure with preserved ejection fraction: A systematic review and meta-analysis of randomized controlled trials | 2018 | Frontiers in Physiology | Not-for-profit | 3.39 | 17 | 2724 |
| 127 | Tian Wang Bu Xin Dan for Insomnia: A Systematic Review of Efficacy and Safety | 2019 | Evidence-based Complementary and Alternative Medicine | Not reported | 1.98 | 14 | 1256 |
| 128 | Chinese herbal medicine Dengzhan Shengmai capsule as adjunctive treatment for ischemic stroke: A systematic review and meta-analysis of randomized clinical trials | 2018 | Complementary Therapies in Medicine | For-profit | 2.08 | 14 | 5206 |
| 129 | Effects of adding Rheum officinale to angiotensin-converting enzyme inhibitors or angiotensin receptor blockers on renal function in patients with chronic renal failure: A meta-analysis of randomized controlled trials | 2018 | Clinical Nephrology | Not-for-profit | 1.35 | 9 | 700 |
| 130 | Motherwort injection for preventing postpartum hemorrhage in women with vaginal delivery: A systematic review and meta-analysis of randomized evidence | 2019 | Evidence-based Complementary and Alternative Medicine | Not-for-profit | 1.98 | 37 | 7887 |
| 131 | Effectiveness and Safety of Oral Cordyceps sinensis on Stable COPD of GOLD Stages 2-3: Systematic Review and Meta-Analysis | 2019 | Evidence-based Complementary and Alternative Medicine | Not-for-profit | 1.98 | 15 | 1238 |
| 132 | Cinobufotalin injection combined with chemotherapy for the treatment of advanced NSCLC in China: A PRISMA-compliant meta-analysis of 29 randomized controlled trials | 2019 | Medicine | No funding | 1.87 | 29 | 2300 |
| 133 | Effectiveness of Chinese herbal medicine for patients with primary insomnia: A PRISMA-compliant meta-analysis | 2019 | Medicine | Not-for-profit | 1.87 | 15 | 1500 |
| 134 | Efficacy and safety of naoshuantong capsule in the treatment of ischemic stroke: A meta-analysis | 2019 | Frontiers in Pharmacology | Not reported | 3.85 | 13 | 1360 |
| 135 | Traditional Chinese medicine for coronary heart disease: Clinical evidence and possible mechanisms | 2019 | Frontiers in Pharmacology | Not-for-profit | 3.85 | 17 | 11732 |
| 136 | Chinese herbal medicine formulas as adjuvant therapy for osteonecrosis of the femoral head: A systematic review and meta-analysis of randomized controlled trials | 2018 | Medicine | Not reported | 2.03 | 23 | 1815 |
| 137 | Efficacy of the Oriental herbal medicine, Jie Yu Dan, for alleviating post-stroke aphasia: A Systematic Review and meta-analysis of randomized clinical trials | 2018 | European Journal of Integrative Medicine | Not-for-profit | 0.70 | 10 | 809 |
| 138 | Chinese Herbal Medicine for Advanced Non-Small-Cell Lung Cancer: A Systematic Review and Meta-Analysis | 2018 | American Journal of Chinese Medicine | Not reported | 3.12 | 64 | 4384 |
| 139 | Chinese herbal medicines on cognitive function and activity of daily living in senior adults with Alzheimer's disease: a systematic review and meta-analysis | 2019 | Integrative Medicine Research | No funding | 0 | 25 | 1855 |
| 140 | The therapeutic effect of Chaihu-Shugan-San in fatty liver disease: A meta-analysis randomized controlled trials | 2018 | International Journal of Clinical and Experimental Medicine | Not-for-profit | 0 | 12 | 1049 |
| 141 | A Review of Danshen Combined with Clopidogrel in the Treatment of Coronary Heart Disease | 2019 | Evidence-based Complementary and Alternative Medicine | Not-for-profit | 1.98 | 22 | 2587 |
| 142 | The Therapeutic Effects of a Traditional Chinese Medicine Formula Wuzi Yanzong Pill for the Treatment of Oligoasthenozoospermia: A Meta-Analysis of Randomized Controlled Trials | 2018 | Evidence-based Complementary and Alternative Medicine | Not reported | 2.06 | 5 | 960 |
| 143 | Efficacy of Chinese herbal medicine in treatment of allergic rhinitis in children: a meta-analysis of 19 randomized controlled trials | 2018 | Journal of International Medical Research | No funding | 1.02 | 19 | 1623 |
| 144 | Suanzaoren formulae for insomnia: Updated clinical evidence and possible mechanisms | 2018 | Frontiers in Pharmacology | Not-for-profit | 3.83 | 13 | 1454 |
| 145 | Clinical Effects and Safety of Tongxieyaofang on Diarrhea Predominant Irritable Bowel Syndrome: A Meta-Analysis of Randomized Trails | 2019 | Evidence-based Complementary and Alternative Medicine | Not reported | 1.98 | 39 | 3062 |
| 146 | Effects of Ginger (Zingiber officinale Roscoe) on Type 2 Diabetes Mellitus and Components of the Metabolic Syndrome: A Systematic Review and Meta-Analysis of Randomized Controlled Trials | 2018 | Evidence-based Complementary and Alternative Medicine | Not reported | 2.06 | 10 | 490 |
| 147 | Traditional Chinese and western medicine for the prevention of deep venous thrombosis after lower extremity orthopedic surgery: a meta-analysis of randomized controlled trials | 2018 | Journal of Orthopaedic Surgery and Research | Not-for-profit | 1.61 | 20 | 1862 |
| 148 | Therapeutic Efficacy of Kangfuxin Liquid Combined with PPIs in Gastric Ulcer | 2019 | Evidence-based Complementary and Alternative Medicine | Not-for-profit | 1.98 | 22 | 2024 |

**Table S4.** Conditions and intervention arms of among included systematic reviews on Chinese herbal medicine

| **ID** | **Name of systematic reviews** | **Type of arms** | | **Condition** |
| --- | --- | --- | --- | --- |
|  |  | **Intervention(s)** | **Control(s)** |  |
| 1 | Efficacy and safety of compound Kushen injection combined with chemotherapy on postoperative Patients with breast cancer: A meta-analysis of randomized controlled trials | Kushen injection + Chemotherapy (not specified) | Conventional treatments (including cyclophosphamide, doxorubicin, 5-fluorouracil, docetaxel, epidoxorubicin, and paclitaxel) | Breast cancer |
| 2 | Zao Ren An Shen for insomnia: a systematic review with meta-analysis | Zao Ren An Shen capsule  Zao Ren An Shen capsule + Conventional treatments (including estazolam, quetiapine, amlodipine, and oxazepam) | Placebo  Conventional treatments (including clonazepam, estazolam, zopiclone, alprazolam, quetiapine, amlodipine, esomezolpine, eszopiclone, and oxazepam) | Insomnia |
| 3 | Add-on effects of Chinese herbal medicine for post-stroke spasticity: A systematic review and meta-analysis | Chinese herbal formulae (including gualouguizhi decoction, modified buyanghuanwu decoction, tongluo jiejing decoction, wenjing shujin capsule, yiqi roujin decoction, buchang naoxintong capsule, roujin decoction, zini wenshen yiqi huoxie decoction, shujin tongluo formula, shujin huoluo xiji, jiejing shujin decoction, jiejing heji, and shaoyaogancao decoction) | Rehabilitation programmes  Rehabilitation programmes + Conventional medications (including baclofen, botulinum toxin, and  tizanidine) | Post-stroke spasticity |
| 4 | Evidence of Astragalus injection combined platinum-based chemotherapy in advanced non-small cell lung cancer patients: A systematic review and meta-analysis | Astragalus injection + Conventional treatments (including mitomycin, vinpocetine, cisplatin, cyclophosphamide, adriamycin, taxol, dexamethasone, irinotecan, etoposide, navelbine, and gemcitabine) | Conventional treatments (including mitomycin, vinpocetine, cisplatin, cyclophosphamide, adriamycin, taxol, dexamethasone, irinotecan, etoposide, navelbine, and gemcitabine) | Advanced non-small-cell lung cancer |
| 5 | Evaluation of the adjunctive effect of Xing Nao Jing Injection for viral encephalitis: A systematic review and meta-analysis of randomized controlled trials | Xingnaojing injection | Placebo  Antiviral drugs (no specified) | Viral encephalitis |
| 6 | Traditional Chinese herbal medicine for vascular dementia | Chinese herbal formulae (including chuanxiongqin, dengzhanhuasu, dengzhanxixin, fufangdanshen, fufanghaishe, gegensu, huangqi, naoan, naomaitai, naoxintong, qingkailing, ruanmailing, shenfu decoction, tongxinluo, xinnaozaizao, yindanxingnaotong, yangxueqingnao, and 25 wei zhenzhu pill)  Chinese herbal formulae + Conventional treatments (including almitrine, aniracetam, cerebroprotein hydrolysate, citicoline, donepezil, ergoloid mesylates, huperzine a, nicergoline, nimodipine, oxiracetam, and piracetam)  Chinese herbal formulae + Conventional treatments + Routine care | Conventional treatments (including almitrine, aniracetam, cerebroprotein hydrolysate, citicoline, donepezil, ergoloid mesylates, huperzine a, nicergoline, nimodipine, oxiracetam, and piracetam)  Conventional treatments + Routine care | Vascular dementia |
| 7 | Qishenyiqi dripping pill improves ventricular remodeling and function in patients with chronic heart failure: A pooled analysis | Qishenyiqi dripping pill + Conventional treatments (not specified) | Conventional treatments (not specified) | Chronic heart failure |
| 8 | Does the Oral Administration of Ginger Reduce Chemotherapy-Induced Nausea and Vomiting?: A Meta-analysis of 10 Randomized Controlled Trials | Ginger root + Conventional treatments (including mixed yogurt, 5-hydroxytryptamine-3 antagonist, dexamethasone, antihistamine, ranitidine, aprepitant, neurokinin 1 receptor antagonist, and metoclopramide) | Conventional treatments (including mixed yogurt, 5-hydroxytryptamine-3 antagonist, dexamethasone, antihistamine, ranitidine, aprepitant, neurokinin 1 receptor antagonist, and metoclopramide) | Chemotherapy-Induced Nausea and Vomiting |
| 9 | Efficacy and safety of Zaoren Anshen capsules in the treatment of insomnia: A meta-analysis of randomized controlled trials | Zaoren Anshen capsule | Conventional treatments (including isalpride, alprazolam, clonazepam, eszopiclone, and zopiclone) | Insomnia |
| 10 | Effects of Shenfu injection on chemotherapy-induced adverse effects and quality of life in patients with advanced non-small cell lung cancer: A systematic review and meta-analysis | Shenfu injection + Conventional treatments (including vinorelbine, cisplatin, gemcitabine hydrochloride, and docetaxel) | Conventional treatments (including vinorelbine, cisplatin, gemcitabine hydrochloride, and docetaxel) | Advanced non-small-cell lung cancer |
| 11 | Efficacy and safety of Xuebijing injection combined with ulinastatin as adjunctive therapy on sepsis: A systematic review and meta-analysis | Xuebijing injection + Ulinastatin | Ulinastatin | Sepsis |
| 12 | Compound Kushen injection combined with platinum-based chemotherapy for stage III/IV non-small cell lung cancer: A meta-analysis of 37 RCTs following the PRISMA guidelines | Compound Kushen injection + Platinum-based chemotherapy (not specified) | Platinum-based chemotherapy (not specified) | Stage III/IV non-small-cell lung cancer |
| 13 | Traditional Chinese medicine yimucao injection combined with western medicine for preventing postpartum hemorrhage after cesarean section: A systematic review and meta-analysis | Yimucao injection + Conventional treatments (including oxytocin, carboprost tromethamine, and misoprostol) | Conventional treatments (including oxytocin, carboprost tromethamine, and misoprostol) | Postpartum haemorrhage |
| 14 | Chinese herbal medicine for myasthenia gravis: A systematic review and meta-analysis | Chinese herbal formulae (including huangqi fufang granules, zhongjiling tablet, fuyuan yiji capsule, yiqi qushi formula, jilikang decoction, bupi qiangli compound, buzhong yiqi decoction, yiqi chushi formula, tan wei capsule, qiangji jianli decoction, and jianjining granules) + Conventional treatments (including pyridostigmine bromide and prednisone) | Placebo  Conventional treatments (including pyridostigmine bromide and prednisone) | Myasthenia gravis |
| 15 | Systematic review and meta-analysis of the efficacy and safety of biqi capsule in rheumatoid arthritis patients | Biqi capsul  Biqi capsule +  Methotrexate | Methotrexate | Rheumatoid arthritis |
| 16 | Chinese herbal medicine for epidermal growth factor receptor inhibitor-induced skin rash in patients with malignancy: An updated meta-analysis of 23 randomized controlled trials | External use of Chinese herbal formulae (including xiaofeng powder,  qingfei liangxe formula, yangfei xiaozhen formula, qingre jiedu qushi formula, pipa qingfei yin, yinqiao powder, puxing jiedu decoction, huangbai powder, jingfangbaidu powder, LG09, jinyinhua, pizen capsule, zhiyang formula, pizhen formula, and wuwei xiaodu yin) | Blank control  Conventional treatments (including hydrocortisone cream, urea ointment, calamine lotion, minocycline, erythromycin ointment, dexamethasone acetate cream, pirimosi ointment, and silicone oil emulsion) | Epidermal growth factor receptor inhibitor-induced skin rash |
| 17 | Effect of Chinese medicine injection for preventing contrast-induced nephropathy after coronary angiography/percutaneous coronary intervention: A systematic review and meta-analysis | Chinese herbal injections (including sodium ferulate, shenxiong glucose, ligustrazine, danhong injection, safflower yellow, astragalus injection, xuebijing injection, salvianolate injection, rhodiola injection, danshen chuanxiongqin injection, and shenkang injection) | Hydration therapy | Coronary artery disease, contrast-induced nephropathy |
| 18 | Efficacy of ginger (zingiber officinale) in ameliorating chemotherapy-induced nausea and vomiting and chemotherapy-related outcomes: a systematic literature review update and meta-analysis | Ginger | Placebo  Intravenous antiemetic  Routine care | Cancer patients receiving chemotherapy |
| 19 | Efficacy and safety of Modified Tongxie Yaofang in diarrhea-predominant irritable bowel syndrome management: A meta-analysis of randomized, positive medicine-controlled trials | Modified Tongxieyaofang  Modified Tongxieyaofang  + Cognitive behavioural therapy | Conventional treatments (including montmorillonite, pinaverium bromide, trimebutine maleate, bacillus licheniformis, loperamide, miyarisam, and glutamine) | Diarrhoea-predominant irritable bowel syndrome |
| 20 | Shexiang Baoxin Pills as an Adjuvant Treatment for Chronic Heart Failure: A System Review and Meta-Analysis | Shexiang Baoxin pills + Conventional treatments (not specified) | Placebo  Conventional treatments (not specified) | Chronic heart failure |
| 21 | Effects of Shengmai injection add-on therapy to chemotherapy in patients with non-small cell lung cancer: a meta-analysis | Shengmai injection + Conventional treatments (including vinorelbine, cisplatin, cyclophosphamide, etoposide, mitomycin, gemcitabine, paclitaxel, carboplatin, adriamycin, docetaxel, and taxinol) | Conventional treatments (including vinorelbine, cisplatin, cyclophosphamide, etoposide, mitomycin, gemcitabine, paclitaxel, carboplatin, adriamycin, docetaxel, and taxinol) | Non-small-cell lung cancer |
| 22 | Efficacy of herbal medicine (Gegen Qinlian Decoction) on ulcerative colitis: A systematic review of randomized controlled trials | Gegen qinlian decoction  Gegen qinlian decoction + Conventional treatments (including methalazine, sulfasalazine, bacillus subtilis, hydrocortisone sodium succinate, and aminosalicylic acid) | Conventional treatments (including methalazine, sulfasalazine, bacillus subtilis, hydrocortisone sodium succinate, and aminosalicylic acid) | Ulcerative colitis |
| 23 | A meta-analysis of the clinical efficacy of TCM decoctions made from formulas in the liuwei dihuang wan categorized formulas in treating diabetic nephropathy proteinuria | Chinese herbal formulae (including jisheng shenqi pill, liuwei dihuang pill, and zhibai dihuang pill) + Conventional treatments (including captopril, benazepril, and valsartan) | Conventional treatments (including captopril, Benazepril, valsartan, enalapril, and telmisartan)  Routine care | Diabetic nephropathy proteinuria |
| 24 | Herbal Formula Modified Buzhong-Yiqi-Tang for Functional Constipation in Adults: A Meta-Analysis of Randomized Controlled Trials | Modifed buzhong yiqi decoction + Conventional treatments (including duphalac, PEG4000, cisapride, mosapride, and Kegel exercises) | Conventional treatments (including duphalac, PEG4000, cisapride, mosapride, and Kegel exercises) | Functional constipation |
| 25 | Safety and Clinical Efficacy of Yangxue Qingnao Granules in the Treatment of Chronic Cerebral Circulation Insufficiency: A Systematic Review and Meta-Analysis | Yangxue qingnao granules + Routine care | Routine care + Conventional treatments (including nimodipine troxerutin, and cinnarizine) | Chronic cerebral circulation insufficiency |
| 26 | Xi huang pills enhance the tumor treatment efficacy when combined with chemotherapy: A meta-analysis and systematic review | Xihuang pill + Conventional treatments (including cyclophosphamide, doxorubicin, vincristine, prednisone, gemcitabine, cisplatin, and 5‑fluorouracil) | Conventional treatments (including cyclophosphamide, doxorubicin, vincristine, prednisone, gemcitabine, cisplatin, and 5‑fluorouracil) | Cancer |
| 27 | Can External Use of Chinese Herbal Medicine Prevent Cumulative Peripheral Neuropathy Induced by Oxaliplatin? A Systematic Literature Review With Meta-analysis | External use of Chinese herbal formulae (including wenyanghuoxuetongluo decoction, huoxuetongjing decoction, wenjinghuoxue decoction, and yangxuewenjingtongluon decoction) | Blank control  Warm water for hand and foot baths | Peripheral neuropathy induced by oxaliplatin in patients with gastrointestinal cancer |
| 28 | Does ginger supplementation lower blood pressure? A systematic review and meta-analysis of clinical trials | Ginger powder  Ginger powder + Black tea  Ginger powder + lifestyle modification training | Placebo  Black tea  Lifestyle modification training | Hypertension |
| 29 | Qingkailing Injection for Treatment | Qingkailing injection  Qingkailing injection + Ertong qingfei solution  Qingkailing injection + Ertong qingfei solution + Xiaoer kechuanning liquid | Ribavirin Injection  Ribavirin injection + Compound guaiacol potassium sulfonate oral solution  Ribavirin injection + Ambroxol hydrochloride injection | Children pneumonia induced by respiratory syncytial virus |
| 30 | Goshajinkigan for reducing chemotherapy-induced peripheral neuropathy: a systematic review and meta-analysis | Goshajinkigan | Placebo  Blank control  Vitamin B12 | Chemotherapy‑induced peripheral neuropathy |
| 31 | Rhubarb combined with trypsin inhibitor for severe acute pancreatitis: A systematic review and meta-analysis | Rhubarb + Conventional treatments (including octreotide, stilamin, ulinastatin, and somatostatin) | Conventional treatments (including octreotide, stilamin, ulinastatin, and somatostatin) | Severe acute pancreatitis |
| 32 | Effect of ginger in the treatment of nausea and vomiting compared with vitamin B6 and placebo during pregnancy: a meta-analysis | Ginger | Placebo  Vitamin B6 | Nausea and vomiting during pregnancy |
| 33 | Dietary ginger as a traditional therapy for blood sugar control in patients with type 2 diabetes mellitus: A systematic review and meta-analysis | Ginger | Conventional treatments (not specified) | Blood sugar control in patients with type 2 diabetes |
| 34 | Efficacy and safety of Shenfu injection for septic shock: A systematic review and meta-analysis of randomized controlled trials | Shenfu injection + Conventional treatments (including norepinephrine, dobutamine, ulinastatin, and dopamine)  Shenfu injection + Routine care  Shenfu injection + Conventional treatments + Routine care | Conventional treatments (including norepinephrine, dobutamine, ulinastatin, and dopamine)  Routine care  Conventional treatments + Routine care | Septic shock |
| 35 | Xuebijing injection for acute organophosphorus pesticide poisoning: A systematic review and meta-Analysis | Xuebijing injection + Conventional treatments (not specified)  Xuebijing injection + Conventional treatments (not specified) + Blood perfusion | Conventional treatments (not specified)  Conventional treatments (not specified) + Blood perfusion | Acute organophosphorus pesticide poisoning |
| 36 | Systematic evaluation of therapeutic efficacy and safety of traditional Chinese medicine injection (TCMI) combined with oxaliplatin-containing chemotherapy in the treatment of colorectal cancer | Chinese herbal injections (including kushen injection, aidi injection, shenqi fuzheng injection, brucei javanica oil emulsion injection, delisheng injection, kang’ai injection, kanglaite injection, xiaoaiping injection, and xiangguduo decoction injection) + Oxaliplatin chemotherapy | Oxaliplatin chemotherapy | Colorectal cancer |
| 37 | Shengmai injection as an adjunctive therapy for the treatment of chronic obstructive pulmonary disease: A systematic review and meta-analysis | Shengmai injection + Oxygen therapy | Oxygen therapy | Chronic obstructive pulmonary disease |
| 38 | Efficacy and safety of Sijunzi decoction for peptic ulcers: A systematic review and meta-analysis | Chinese herbal formulae (including sijunzi decoction, modified sijunzi decoction, modified sijunzi ulcer-healing decoction, and sijunzi mixture)  Chinese herbal formulae + Conventional treatments (including omeprazole, amoxicillin, clarithromycin, cimetidine, sucralfate, metronidazole, bismuth pectin, ranitidine, levofloxacin, furazolidone, and pantoprazole) | Conventional treatments (including omeprazole, amoxicillin, clarithromycin, cimetidine, sucralfate, metronidazole, bismuth pectin, ranitidine, levofloxacin, furazolidone, and pantoprazole) | Peptic ulcers |
| 39 | Kanglaite injection plus platinum-based chemotherapy for stage III/IV non-small cell lung cancer: A meta-analysis of 27 RCTs | Kanglaite injection + Platinum-based chemotherapy | Platinum-based chemotherapy | Stage III/IV non-small-cell lung cancer |
| 40 | The effects of ginger supplementation on markers of inflammatory and oxidative stress: A systematic review and meta-analysis of clinical trials | Ginger | Not reported | Inflammatory and oxidative stress markers |
| 41 | Does Adjuvant Treatment with Chinese Herbal Medicine to Antidiabetic Agents Have Additional Benefits in Patients with Type 2 Diabetes? A System Review and Meta-Analysis of Randomized Controlled Trials | Chinese herbal formulae (including berberine, TM81, xiaoke pills, jinlida, jianyutangkang, gegenqinlian, tangke, tangminling pills, tianqijiang decoction, and liuweidihuang combined with ginkgo leaf) + Conventional treatments (including metformin and glibenclamide) | Placebo  Conventional treatments (including metformin and glibenclamide) | Type 2 diabetes |
| 42 | Efficacy and Safety of Xiaoyao Formula as an Adjuvant Treatment for Post-Stroke Depression: A Meta-Analysis | Xiaoyao pill + Conventional treatments (including fluoxertine, deanxit, venlafaxine, and escitalopram) | Conventional treatments (including fluoxertine, deanxit, venlafaxine, and escitalopram) | Post-stroke depression |
| 43 | Danggui-Shaoyao-San for dementia: A PRISMA-compliant systematic review and meta-analysis | Danggui shaoyaosan  Danggui shaoyaosan  + Acupuncture  Danggui shaoyaosan  + Electroacupuncture | Vitamin E  Acupuncture  Electroacupuncture  Conventional treatments (including nimodipine, huperzine A, piracetam, duxil, citicoline, and oxiracetam) | Dementia |
| 44 | Xiao Chai Hu Tang, a herbal medicine, for chronic hepatitis B | Xiaochaihu decoction | Blank control | Chronic hepatitis B |
| 45 | Efficacy and safety of Sihogayonggolmoryeo-tang (Saikokaryukotsuboreito, Chai-Hu-Jia-Long-Gu-Mu-Li-Tang) for post-stroke depression: A systematic review and meta-analysis | Chaihu jia longgumuli decoction  Chaihu jia longgumuli decoction + Routine care  Chaihu jia longgumuli decoction + Routine care + Psychotherapy  Chaihu jia longgumuli decoction + Routine care + Conventional treatments (including flupentixol and melitracen) | Conventional treatments (including smitriptyline, flupentixol and melitracen)  Conventional treatments + Routine care | Post-stroke depression |
| 46 | Herbal medicine (Gegen-decoction) for treating cervical spondylosis: A systematic review and meta-analysis of randomized controlled trials | Modified gegen decoction  Modified gegen decoction  + Acupuncture  Modified gegen decoction  + Conventional treatments (including ibuprofen, chlorzoxazone, and flunarizine) | Conventional treatments (including ,ecobalamin, ibuprofen, chlorzoxazone, and flunarizine)  Acupuncture | Cervical spondylosis |
| 47 | Herbal medicine (Hyeolbuchukeo-tang or Xuefu Zhuyu decoction) for treating primary dysmenorrhea: A systematic review and meta-analysis of randomized controlled trials | Xuefu zhuyu decoction  Xuefu zhuyu decoction + Conventional treatments (including fenbid, andyzanol, thiamine, indometacin, naproxen, ibuprofen, and diclofenac) | Conventional treatments (including fenbid, andyzanol, thiamine, indometacin, naproxen, ibuprofen, and diclofenac) | Primary dysmenorrhea |
| 48 | Efficacy and Safety of Chinese Herbs for the Prevention of the Risk of Renal Damage in Henoch-Schonlein Purpura in Children: Meta-Analysis of Randomized Controlled Trials and GRADE Evaluation | Chinese herbal formulae (including xijiao dihuang decoction, modifed huaban decoction, wuteng granules, kunxian granules, liangxue xiaodian decoction, huaiqihuang granules, shitan decoction, xiaofeng formula, zidian zhenxiao granules, yinqiao formula, zidan particles, and kangmin xiaodian decoction) + Conventional treatments (not specified) | Conventional treatments (not specified) | Renal damage in Henoch-Schonlein Purpura |
| 49 | Efficacy and safety of Xuebijing injection (a Chinese patent) for sepsis: A meta-analysis of randomized controlled trials | Xuebijing injection + Routine care | Routine care | Sepsis |
| 50 | Efficacy and safety of TCM Yangxin Anshen Therapy for insomnia: A systematic review and meta-analysis | Yangxin anshen decoction | Placebo | Insomnia |
| 51 | Gardenia fructus antidepressant formula for depression in diabetes patients: A systematic review and meta-analysis | Gardeniae fructus preparations (including zishen qinggan decoction, shuyu qingxiao decoction, jiawei danzhixiaoyao powder, xiaoke jieyu decoction, jieyu decoction, jiaweixiaoyao pill, jieyu anshen decoction, yangyin jieyu decoction, and jiawei xiaoyao powder)  Gardeniae fructus preparations + Conventional treatments (including fluoxetine and sertraline) | Blank control  Conventional treatments (including fluoxetine and sertraline) | Depression in diabetes patients |
| 52 | Twelve Chinese herbal preparations for the treatment of depression or depressive symptoms in cancer patients: a systematic review and meta-analysis of randomized controlled trials | Chinese herbal formulae (including Xuefuzhuyu decoction, Yangfeixiaojijieyu decoction, Danzhixiaoyao decoction, Chaihushugan decoction, Tongyou decoction, Ganmaidazao decoction, Xiaoyao decoction, Shuganjieyu capsule, Suanzaorenjialongmu decoction, Shuganjieyuhuaji decoction, Chaihujialonggumuli decoction, Banxiahoupo decoction, and liujunzi decoction) | Blank control  Conventional treatments (including fluoxetine, alprazolam, flupenthixol, and melitracen)  Psychotherapy | Depression or depressive symptoms |
| 53 | Chinese herbal formulae for the treatment of menopausal hot flushes: A systematic review and meta-analysis | Chinese herbal formulae (including kunbao pill honey pill, kuntai capsule, dingkun dan, modified danzhi xiaoyao powder, heyan kuntai capsule, guizhi fuling granules, erzhi granules, MF101, danggui buxue decoction, zhimu 14 capsule, modified erxian decoction, zhibai dihuang pill, modified qing e formula, and gengnianan granules) | Placebo  Conventional treatments (including oestrogen and progesterone) | Menopausal hot flushes |
| 54 | Potential effectiveness of Chinese patent medicine Tongxinluo capsule for secondary prevention after acute myocardial infarction: A systematic review and meta-analysis of randomized controlled trials | Tongxinluo capsule + Conventional treatments (including trimetazidine, atorvastatin, metoprolol, urokinase, and simvastatin) | Placebo  Conventional treatments (including trimetazidine, atorvastatin, metoprolol, urokinase, and simvastatin) | Acute myocardial infarction |
| 55 | Xiyanping plus Azithromycin Chemotherapy in Pediatric Patients with Mycoplasma pneumoniae Pneumonia: A Systematic Review and Meta-Analysis of Efficacy and Safety | Xiyanping + Azithromycin chemotherapy | Blank control  Interventions other than azithromycin chemotherapy (not specified) | Paediatric patients with mycoplasma pneumoniae pneumonia |
| 56 | Effectiveness of traditional Chinese medicine Jinlida granules as an add-on therapy for type 2 diabetes: A system review and meta-analysis of randomized controlled trials | Jinlida + Conventional treatments (including metformin, repaglinide, pioglitazone, gliclazide, and sitagliptin) | Placebo  Conventional treatments (including metformin, repaglinide, pioglitazone, gliclazide, and sitagliptin) | Type 2 diabetes |
| 57 | Huangqi Guizhi Wuwu Decoction for treating cervical radiculopathy: A systematic review and meta-analysis of randomized controlled trials | Modified huangqi guizhi wuwu gecoction  Modified huangqi guizhi wuwu gecoction + Manipulation  Modified huangqi guizhi wuwu gecoction + Acupuncture | Mecobalamin  Jing fukang granules  Manipulation  Acupuncture | Cervical radiculopathy |
| 58 | Angong Niuhuang Pill as adjuvant therapy for treating acute cerebral infarction and intracerebral hemorrhage: A meta-analysis of randomized controlled trials | Niuhuang Pill + Conventional treatments (including lower intracranial pressure, control blood pressure, prevent upper gastrointestinal bleeding and pulmonary infection, mannitol, captopril, correct water-electrolyte and acid-base balance, aspirin, nimodipine, citicoline, naloxone, oxiracetam injection, ganglioside, and edaravon) | Conventional treatments (including lower intracranial pressure, control blood pressure, prevent upper gastrointestinal bleeding and pulmonary infection, mannitol, captopril, correct water-electrolyte and acid-base balance, aspirin, nimodipine, citicoline, naloxone, oxiracetam injection, ganglioside, and edaravon) | Acute cerebral infarction and intracerebral haemorrhage |
| 59 | Efficacy and safety of Kanglaite injection combined with radiochemotherapy in the treatment of advanced pancreatic cancer: A PRISMA-compliant meta-analysis | Kanglaite + Conventional treatments (including gemcitabine, gimeracil, oteracil porassium, 3-dimensional conformal radiotherapy, cisplatin, fluorouracil, and g-ray stereotactic body radiation therapy) | Conventional treatments (including gemcitabine, gimeracil, oteracil porassium, 3-dimensional conformal radiotherapy, cisplatin, fluorouracil, and g-ray stereotactic body radiation therapy) | Advanced pancreatic cancer |
| 60 | Effects of Aidi Injection with Western Medical Therapies on Quality of Life for Patients with Primary Liver Cancer: A Systematic Review and Meta-Analysis | Aidi Injection + Conventional treatments (not specified) | Conventional treatments (not specified) | Primary liver cancer |
| 61 | Traditional Chinese medicine for Bradyarrhythmia: Evidence and potential mechanisms | Xinbao pill | Placebo  Routine care | Bradyarrhythmia |
| 62 | Shenqi Fuzheng Injection in the Treatment of Breast Cancer: A Meta-analysis of Randomized Controlled Trials | Shenqi fuzheng injection + Conventional treatments (including cyclophosphamide, doxorubicin, 5-fluorouracil, pirarubicin, epirubicin, anthracyclines, navelbine, methotrexate, capecitabine, paclitaxel, docetaxel, gemcitabine, cisplatin, and mitomycin) | Conventional treatments (including cyclophosphamide, doxorubicin, 5-fluorouracil, pirarubicin, epirubicin, anthracyclines, navelbine, methotrexate, capecitabine, paclitaxel, docetaxel, gemcitabine, cisplatin, and mitomycin) | Breast cancer |
| 63 | Clinical Efficacy of Jinshuibao Capsules Combined with Angiotensin Receptor Blockers in Patients with Early Diabetic Nephropathy: A Meta-Analysis of Randomized Controlled Trials | Jinshuibao capsule + Conventional treatments (including valsartan, irbesartan, candesartan, telmisartan, losartan, and Olmesartan) | Conventional treatments (including valsartan, irbesartan, candesartan, telmisartan, losartan, and Olmesartan) | Early diabetic nephropathy |
| 64 | Sanjin tablet combined with antibiotics for treating patients with acute lower urinary tract infections: A meta-analysis and GRADE evidence profile | Sanjin tablet + Conventional treatments (including levofloxacin, gatifloxacin, and oxyfluoxacin) | Placebo  Conventional treatments (including levofloxacin, gatifloxacin, and oxyfluoxacin) | Acute lower urinary tract infections |
| 65 | Tongxinluo Capsule for Cardiac Syndrome X: A Systematic Review and Meta-Analysis | Tongxinluo capsule  Tongxinluo capsule + Conventional treatments (including nitrates, diltiazem, and metoprolol) | Placebo  Conventional treatments (including nitrates, diltiazem, and metoprolol) | Cardiac syndrome X |
| 66 | Oral Chinese Herbal Medicine for Heart Failure with Preserved Ejection Fraction: A Meta-Analysis | Chinese herbal formulae (including qili qiangxin capsule and shexiang baoxin pill)  Chinese herbal formulae + Conventional treatments (not specified) | Placebo  Blank control  Conventional treatments (not specified) | Heart failure with preserved ejection fraction |
| 67 | Efficacy and safety of motherwort injection add-on therapy to carboprost tromethamine for prevention of post-partum blood loss: A meta-analysis of randomized controlled trials | Motherwort + Carboprost tromethamine | Carboprost tromethamine | Post-partum haemorrhage |
| 68 | Add-on effect of Guizhi Fuling formula to mifepristone for endometriosis: A meta-analysis of randomized controlled trials | Guizhi fuling capsule + Mifepristone  Guizhi fuling capsule + Mifepristone + Laparoscopy | Mifepristone  Mifepristone + Laparoscopy | Endometriosis |
| 69 | Effects of ginseng supplementation on selected markers of inflammation: A systematic review and meta-analysis | Ginseng | Not reported | Inflammation |
| 70 | A systematic review on Chinese herbal treatment for radiotherapy-induced xerostomia in head and neck cancer patients | Chinese herbal formulae (including yunujian formula, shenqi fanghou formula, yangyin jiedu decoction, zhong jie feng, shengjin formula, xuanmai zengye huadu decoction, qingre yangyin shengjin formula, and qingfei yangwei formula) + Radiotherapy  Chinese herbal formulae + Radiotherapy + Chemotherapy | Radiotherapy  Chemotherapy + Radiotherapy | Radiotherapy-induced xerostomia in head and neck cancer patients |
| 71 | Traditional Chinese Patent Medicine for Treating Impaired Glucose Tolerance: A Systematic Review and Meta-Analysis of Randomized Controlled Trials | Chinese herbal formulae (including tangzhiping granules, shenzhu tiaopi granules, shenqijiang decoction, liuwei dihuang pill, xiaoke huayu tablet, jinqijiang decoction, xiaoke jianpi capsule, xiaoke yuye capsule, tianqi capsule, tangyikang pill, jianpi qinghua granules, and tianmai xiaoke tablet) + Lifestyle modification | Placebo  Lifestyle modification  Placebo + Lifestyle modification | Impaired glucose tolerance |
| 72 | Clinical evidence on the effects of saffron (Crocus sativus L.) on cardiovascular risk factors: A systematic review meta-analysis | Saffron  Saffron + Tea  Saffron + olanzapine | Placebo  Tea  Olanzapine | Cardiovascular disease |
| 73 | The effect of ginger supplementation on lipid profile: A systematic review and meta-analysis of clinical trials | Ginger | Blank control  Placebo | Cardiovascular disease |
| 74 | KangFuXin Liquid in the Treatment of Diabetic Foot Ulcer: A Systematic Review and Meta-Analysis | kangfuxin liquid  kangfuxin liquid + Insulin | Insulin  Antibiotics (not specified)  Nutritional support  Vasodilator  Insulin + Antibiotics + Nutritional support + Vasodilator | Diabetic foot ulcer |
| 75 | Effects of saffron (Crocus sativus) on sexual dysfunction among men and women: A systematic review and meta-analysis | Saffron capsule | Placebo | Sexual dysfunction |
| 76 | Efficacy of Suxiao Jiuxin Pill on Coronary Heart Disease: A Meta-Analysis of Randomized Controlled Trials | Suxiao jiuxin pill | Placebo  Conventional treatments (including shexiang baoxin pills, nitroglycerin, isosorbide dinitrate, huoxin pills, xinkeshu capsule, glucose-insulin-potassium therapy, hesu pills, and tongxinluo pills) | Coronary heart disease |
| 77 | Chinese herbal medicines for the treatment of non-structural abnormal uterine bleeding in perimenopause: A systematic review and a meta-analysis | Chinese herbal formulae (including zhixuefang, shenqishixiao powder, renshen yangrong decoction, baohua decoction, guipi pill,  zibu ganshen decoction, gongxueting formula, ankun decoction, xuejianchou pill, bushen huoxue formula, jianpi guchong decoction, yiqi jianpi guchong decoction, qigui yimu decoction, wuxian zhixue granules, and shenghua decoction) | Conventional treatments (including  oestrogen and progesterone) | Perimenopause |
| 78 | Chinese herbal medicines of supplementing Qi and nourishing Yin combined with chemotherapy for non-small cell lung cancer: A meta-analysis and systematic review | Chinese herbal formulae (including yiqi yangyin sugan decoction, yiqi yangyin yiliu decoction, yiqi yangyin jiedu decoction, yiqiyangyin fuzhengguben decoction) + Conventional treatments (including cisplatin, vinorelbine, gemcitabine, docetaxel, cyclophosphamide, adriamycin, and platinum) | Conventional treatments (including cisplatin, vinorelbine, gemcitabine, docetaxel, cyclophosphamide, adriamycin, and platinum) | Non-small-cell lung cancer |
| 79 | Corn silk tea for hypertension: A systematic review and meta-analysis of randomized controlled trials | Corn silk + Conventional treatments (including nifedipine, enalapril, and folic acid) | Conventional treatments (including nifedipine, enalapril, and folic acid) | High blood pressure |
| 80 | Corn silk decoction for blood lipid in patients with angina pectoris: A systematic review and meta-analysis | Corn silk | Conventional treatments (not specified) | Angina pectoris |
| 81 | Chinese herbal medicine for headache: A systematic review and meta-analysis of high-quality randomized controlled trials | Chinese herbal formulae (including zhengtian pill, chuanxiong dingtong granules, duliang soft capsule, wuzhuyu decoction, qinggan xiehuo zhitong decoction, yangxue qingnao granules, yangxue pinggan granules, among others) | Placebo  Conventional treatments (including flunarizine, ibuprofen, oxcarbazepine, nimodipine, and deanxit) | Headache |
| 82 | Treatment of depression with Chai Hu Shu Gan San: a systematic review and meta-analysis of 42 randomized controlled trials | Chaihu shugan powder  Chaihu shugan powder + Conventional treatments (including fluoxetine, venlafaxine, mirtazapine, amitriptyline, citalopram, escitalopram, deanxit, sertraline, and duloxetine) | Chaihu shugan powder + Conventional treatments (including fluoxetine, venlafaxine, mirtazapine, amitriptyline, citalopram, escitalopram, deanxit, sertraline, and duloxetine) | Depression |
| 83 | Effectiveness and safety of Chinese herbal medicine for pediatric adenoid hypertrophy: A meta-analysis | Chinese herbal formulae (including huiyanzhuyu decoction, baihujiarenshen decoction, qingfeitongqiao formula, shunxi decoction, cangerzi powder, huatansanjie decoction, jianpi huashi sanjie decoction, jianpihuayu formula, and xintong granules) | Conventional treatments (including mometasone, clarithromycin, roxithromycin, budesonide, pidotimod, chloropheniramine, and ketotifen) | Paediatric adenoid hypertrophy |
| 84 | Randomized Controlled Trials of Tianma Gouteng Decoction Combined with Nifedipine in the Treatment of Primary Hypertension: A Systematic Review and Meta-Analysis | Tianma gouteng decoction + Nifedipine | Nifedipine | Primary hypertension |
| 85 | Traditional Chinese medicine injections in the treatment of diabetic foot: A systematic review and meta-analysis | Chinese herbal injections (including ginkgo biloba extract injection, erigeron breviscapus extract injection, panax notoginsenosides injection, compound salvia miltiorrhiza injection, and danhong injection) + Routine care  Chinese herbal injections + Conventional treatments (including alprostadil, anisodamine, ozagrel, pancreatic kininogenase, and mecobalamin) + Routine care | Conventional treatments (including alprostadil, anisodamine, ozagrel, pancreatic kininogenase, and mecobalamin)  Routine care  Conventional treatments + Routine care | Diabetic foot |
| 86 | Efficacy and Safety Evaluation of Taohong Siwu Decoction for Patients with Angina Pectoris: A Meta-Analysis of Randomized Controlled Trials | Taohong siwu decoction + Conventional treatments (not specified) | Conventional treatments (not specified) | Cardiovascular disease |
| 87 | The Efficacy of Saffron in the Treatment of Mild to Moderate Depression: A Meta-analysis | Saffron | Placebo  Conventional treatments (including fluoxetine, citalopram, and imipramine) | Depression |
| 88 | The Effects of a Fixed Combination of Berberis aristata and Silybum marianum on Dyslipidaemia - A Meta-analysis and Systematic Review | Berberol | Placebo | Dyslipidaemia |
| 89 | Efficacy and safety of Chinese herbal medicine for primary intracerebral hemorrhage: A systematic review of randomized controlled trials | Chinese herbal formulae (including herbs of radix et rhizoma rhei, radix notoginseng, panax notoginseng, rhizoma ligustici chuanxiong, radix paeoniae rubra, rhizoma acori tatarinowii, radix curcumae, fructus gardeniae, semen persicae, flos carthami, radix glycyrrhizae, radix angelicae, among others) + Conventional treatments (not specified) | Placebo  Conventional treatments (not specified) | Primary intracerebral haemorrhage |
| 90 | Aidi injection plus platinum-based chemotherapy for stage IIIB/IV non-small cell lung cancer: A meta-analysis of 42 RCTs following the PRISMA guidelines | Aidi injection + Platinum-based chemotherapy | Platinum-based chemotherapy | Stage IIIB/IV non-small-cell lung cancer |
| 91 | Adjuvant Therapy of Oral Chinese Herbal Medicine for Menopausal Depression: A Systematic Review and Meta-Analysis | Chinese herbal formulae (including the herbs of radix bupleuri, and radix paeoniae alba) + Conventional treatments (including paroxetine, fluoxetine, deanxit, and venlafaxine, estradiol, and tibolone) | Conventional treatments (including paroxetine, fluoxetine, deanxit, and venlafaxine, estradiol, and tibolone) | Menopausal depression |
| 92 | Efficacy and safety of Chinese herbal medicine on ovarian cancer after reduction surgery and adjuvant chemotherapy: A systematic review and meta-analysis | Chinese herbal formulae (including shenlingbaizhu decoction, guizhifuling capsules, among others) + Conventional treatments (including docetaxel, cisplatin, taxol, carboplatin)  Chinese herbal formulae + Routine care | Conventional treatments (including docetaxel, cisplatin, taxol, carboplatin)  Routine care | Ovarian cancer |
| 93 | The efficacy of Xue Fu Zhu Yu prescription for hyperlipidemia: A meta-analysis of randomized controlled trials | Xuefu zhuyu decoction  Xuefu zhuyu decoction + Simvastatin | Placebo  Conventional treatments (including simvastatin, hexanicit, gemfibrozil, and polysaccharide sulfate) | Hyperlipidaemia |
| 94 | Danshen Formulae for Cancer: A Systematic Review and Meta-Analysis of High-Quality Randomized Controlled Trials | Salvia Miltiorrhiza preparations (including danshen formula, jiedu huayu formula, zilongjin tablet, aixiao ruangan decoction, jianpi kangai mixture, fuzhengkangai decoction, and xidan decoction) + Conventional treatments (including vinorelbine, cisplatin, dexamethasone, daunorubicin, cytarabine, and paclitaxel)  Salvia Miltiorrhiza preparations + Radiotherapy | Blank control  Radiotherapy  Conventional treatments (including vinorelbine, cisplatin, dexamethasone, daunorubicin, cytarabine, and paclitaxel) | Cancer |
| 95 | The effects of Chinese herbal medicines for treating diabetic foot ulcers: A systematic review of 49 randomized controlled trials | Chinese herbal formulae (including mahuang tincture, wenjing sanhan muzu formula, buyang huanwu decoction, simiao yongan decoction, raw honey, yunnan baiyao, tangzu mixture, buzhong yiqi decoction, shengji yuhong ointment, bingmi ointment, yinlian decoction, sanhuang decoction, xiangpi shengji ointment, jingjie lianqiao decoction, shengji powder, among others) + Conventional treatments (not specified)  Chinese herbal formulae + Routine care | Placebo  Conventional treatments (not specified)  Routine care | Diabetic foot ulcers |
| 96 | Erxian decoction, a Chinese herbal formula, for menopausal syndrome: An updated systematic review | Erxian decoction  Erxian decoction + Other Chinese herbel formulae (including jiaotai pill, erzhi pill, ganmaidazao decoction, and xiaoyaosan)  Erxian decoction + Suanzaoren decoction + acupuncture + Moving cupping | Placebo  Conventional treatments (including oestrogen and progesterone) | Menopausal syndrome |
| 97 | Efficacy and safety of Chinese herbal medicine for depression: A systematic review and meta-analysis of randomized controlled trials | Chinese herbal formulae (including herbs of radix bupleuri, hypericum perfoliatum, radix paeoniae alba, radix ginseng, poria, rhizoma anemarrhenae, bulbus lilii, radix astragali, arillus longan, rhizoma cyperi, among others)  Chinese herbal formulae + Conventional treatments (not specified) | Placebo  Conventional treatments (not specified) | Depression |
| 98 | Chinese herbal medicine combined with tadalafil for erectile dysfunction: a systematic review and meta-analysis | Chinese herbal formulae (including erdibiejia decoction, shisanwei ziyinzhuangyang capsule, baji capsule, compound xuanju capsule, congrong yishen capsule, shugan yiyang capsule, and hongjingtian I decoction) + Tadalafil | Tadalafil | Erectile dysfunction |
| 99 | Acupoint herbal patching during Sanfu Days on reducing frequency of acute asthma attack in children: A systematic review and meta-analysis | Acupoint herbal patching (including herbs of white mustard seed, radix kansui, and rhizoma corydalis) + Conventional treatments (including glucocorticoid, seretide, montelukast, budesonide, and salbutamol) | Conventional treatments (including glucocorticoid, seretide, montelukast, budesonide, and salbutamol) | Acute asthma attack |
| 100 | Kanglaite Injection Combined with Chemotherapy versus Chemotherapy Alone for the Improvement of Clinical Efficacy and Immune Function in Patients with Advanced Non-Small-Cell Lung Cancer: A Systematic Review and Meta-Analysis | Kanglaite injection + Conventional treatments (including gemcitabine, platinum, navelbine, docetaxel, and taxol) | Conventional treatments (including gemcitabine, platinum, navelbine, docetaxel, and taxol) | Advanced non-small-cell lung cancer |
| 101 | Effect of Zhizhu Kuanzhong Capsules on Treatment of Functional Dyspepsia: A Meta-Analysis of Randomized Controlled Trials | Zhizhu kuanzhong capsule  Zhizhu kuanzhong capsule + Conventional treatments (including esomeprazole, amitriptyline, clebopride, pancreatin enteric coated tablet, mosapride, omeprazole, flupentixol, melitrance, domperidone, lansoprazole, alprazolam, trimebutine, and mianserin) | Conventional treatments (including esomeprazole, amitriptyline, clebopride, pancreatin enteric coated tablet, mosapride, omeprazole, flupentixol, melitrance, domperidone, lansoprazole, alprazolam, trimebutine, and mianserin) | Functional dyspepsia |
| 102 | Chinese Herbal Medicines for Restenosis After Percutaneous Coronary Intervention: A Meta-Analysis of Randomized Controlled Trials | Chinese herbal formulae (including shenhong huazhuo tongluo granules, XS0601, xintong II, zintong III, xiongshao capsule, yixin tongmai decoction, tongxinluo capsule, and huxinkang tablet) + Conventional treatments (including aspirin, clopidogrel, ticlopidine, atorvastatin, and simvastatin) | Placebo  Routine care  Conventional treatments (including aspirin, clopidogrel, ticlopidine, atorvastatin, and simvastatin) | Restenosis |
| 103 | Efficacy and Safety of Xiao Ai Ping Injection Combined with Chemotherapy in Advanced Gastric Cancer: A Systematic Review and Meta-Analysis | Xiaoaiping injection + Conventional treatments (including capecitabine, oxaliplatin, tegafur, calcium folinate, 5-fluorouracil, irinotecan, paclitaxel, and cisplatin) | Conventional treatments (including capecitabine, oxaliplatin, tegafur, calcium folinate, 5-fluorouracil, irinotecan, paclitaxel, and cisplatin) | Advanced gastric cancer |
| 104 | Yinzhihuang oral liquid combined with phototherapy for neonatal jaundice: a systematic review and meta-analysis of randomized clinical trials | Yinzhihuang oral liquid + Phototherapy | Phototherapy | Neonatal jaundice |
| 105 | Meta analysis of clinical efficacy of traditional Chinese medicine in the treatment of aplastic anemia | Liuwei dihuang pill + Compound zaofan pill | Androgen | Aplastic anaemia |
| 106 | Curative efficacy and safety of traditional Chinese medicine xuebijing injections combined with ulinastatin for treating sepsis in the Chinese population: A meta-analysis | Xuebijing + Ulinastatin + Routine care | Routine care | Sepsis |
| 107 | Clinical efficacy and safety of aidi injection combination with vinorelbine and cisplatin for advanced non-small-cell lung carcinoma: A systematic review and meta-analysis of 54 randomized controlled trials | Aidi injection + Vinorelbine + Cisplatin | Vinorelbine + Cisplatin | Advanced non-small-cell lung cancer |
| 108 | Clinical efficacy and safety of Aidi injection plus paclitaxel-based chemotherapy for advanced non-small cell lung cancer: A meta-analysis of 31 randomized controlled trials following the PRISMA guidelines | Aidi injection + Conventional treatments (including paclitaxel, cisplatin, oxaliplatin, and carboplatin) | Conventional treatments (including paclitaxel, cisplatin, oxaliplatin, and carboplatin) | Advanced non-small-cell lung cancer |
| 109 | Can Aidi injection improve overall survival in patients with non-small cell lung cancer? A systematic review and meta-analysis of 25 randomized controlled trials | Aidi injection + Radiotherapy  Aidi injection + Conventional treatments (including navelbine, paclitaxel, cisplatin, gemcitabine, oxaliplatin, docetaxel and carboplatin)  Aidi injection + Bronchial arterial chemoembolisation | Radiotherapy  Conventional treatments (including navelbine, paclitaxel, cisplatin, gemcitabine, oxaliplatin, docetaxel and carboplatin)  Bronchial arterial chemoembolisation | Non-small-cell lung cancer |
| 110 | Yizhi decoction as a therapy for vascular dementia: A meta-analysis | Yizhi decoction | Placebo | Vascular dementia |
| 111 | Clinical Efficacy and Safety of Tanreqing Injection for Pulmonary Infection in Patients with Tuberculosis: A Meta-Analysis | Tanreqing injection  Tanreqing injection + Antibiotics (including cefuroxime, cefoperazone, ceftriaxone, cefotaxime, ceftezole, isoniazid, rifampicin, pyrazinamide, ethambutol, streptomycin, and levofloxacin) | Antibiotics (including cefuroxime, cefoperazone, ceftriaxone, cefotaxime, ceftezole, isoniazid, rifampicin, pyrazinamide, ethambutol, streptomycin, and levofloxacin) | Pulmonary infection with tuberculosis |
| 112 | Efficacy and safety of Chinese herbal medicine Xiao Yao San in hypertension: A systematic review and meta-analysis | Xiaoyao powder  Xiaoyao powder + Conventional treatments (including nifedipine, doxepin, amlodipine besylate, lorazepam, fluoxetine, dihydrochlorothiazide, maprotiline, buspirone hydrochloride, valsartan, metoprolol, enalapril, and oryzanol)  Xiaoyao powder + Lifestyle modifications (including exercising, controlling diet, quitting smoking, and limiting alcohol) | Conventional treatments (including nifedipine, doxepin, amlodipine besylate, lorazepam, fluoxetine, dihydrochlorothiazide, maprotiline, buspirone hydrochloride, valsartan, metoprolol, enalapril, and oryzanol)  Lifestyle modifications (including exercising, controlling diet, quitting smoking, and limiting alcohol) | Hypertension |
| 113 | Efficacy and safety of Chinese herbal medicine for patients with postmenopausal hypertension: A systematic review and meta-analysis | Chinese herbal formulae (including modified erxian decoction, xuzao gengping powder, songling xuemaikang capsule, zhengan xifeng decoction, banxia baizhu tianma decoction, liu wei dihuang pill, xiaoyao pill, suanzaoren decoction, wendan decoction, and wuling capsule) + Conventional treatments (including nitrendipine, benazepril, nifedipine, losartan, telmisartan, valsartan, levamlodipine, felodipine, amlodipine, captopril, fosinopril, oryzanol, enalapril, perindopril, irbesartan, telmisartan, and hydrochlorothiazide) | Conventional treatments (including nitrendipine, benazepril, nifedipine, losartan, telmisartan, valsartan, levamlodipine, felodipine, amlodipine, captopril, fosinopril, oryzanol, enalapril, perindopril, irbesartan, telmisartan, and hydrochlorothiazide) | Postmenopausal hypertension |
| 114 | Therapeutic efficacy and safety of traditional Chinese medicine classic herbal formula Longdanxiegan decoction for hypertension: A systematic review and meta-analysis | Longdanxiegan decoction  Longdanxiegan decoction + Conventional treatments (including hydrochlorothiazide, nifedipine, sodium nitroprusside, amlodipine, candesartan, captopril, and metoprolol) | Conventional treatments (including hydrochlorothiazide, nifedipine, sodium nitroprusside, amlodipine, candesartan, captopril, and metoprolol) | Hypertension |
| 115 | Efficacy and safety of Chinese patent medicine (Jinlong capsule) in the treatment of advanced hepatocellular carcinoma: a meta-analysis | Jinlong capsule + Transcatheter arterial chemoembolisation (including pirarubicin, fluorouracil, mitomycin C, epirubicin, cisplatin, calcium levofolinate, carboplatin, hydroxycamptothecin, oxaliplatin, and gemcitabine)  Jinlong capsule + Radiofrequency ablation + Percutaneous ethanol injection  Jinlong capsule + Routine care  Jinlong capsule + Radiotherapy  Jinlong capsule + Surgery + Chemotherapy | Transcatheter arterial chemoembolisation (including pirarubicin, fluorouracil, mitomycin C, epirubicin, cisplatin, calcium levofolinate, carboplatin, hydroxycamptothecin, oxaliplatin, and gemcitabine)  Radiofrequency ablation + Percutaneous ethanol injection  Routine care  Radiotherapy  Surgery + Chemotherapy | Advanced hepatocellular carcinoma |
| 116 | Chinese herbal medicine for Wilson's disease: A systematic review and meta-analysis | Chinese herbal formulae (including gandouling tablet, chaihuang gandou powder, shugan lidan paidu decoction, dahuang gandou decoction, and ruanjian syrup) + Conventional treatments (including sodium di-mercaptopropanesulfonate, dimercaptosuccinate acid, glutathione, penicillamine, and zincsulfate) | Conventional treatments (including sodium di-mercaptopropanesulfonate, dimercaptosuccinate acid, glutathione, penicillamine, and zincsulfate) | Wilson's disease |
| 117 | Role of Xingnaojing combined with naloxone in treating intracerebral haemorrhage: A systematic review and meta-analysis of randomized controlled trials | Xingnaojing + Naloxone | Naloxone | Intracerebral haemorrhage |
| 118 | Chinese herbal bath therapy for the treatment of uremic pruritus: meta-analysis of randomized controlled trials | Chinese herbs for bath therapy (including fructus kochiae scopariae, radix sophorae flavescentis, cortex dictamni, periostracum cicadae, fructus cnidii, spina gleditsiae, fructus tribuli terrestris, among others) + Conventional treatments (including haemodialysis and haemoperfusion) | Shan Chinese herbal bath therapy  Shan Chinese herbal bath therapy + Haemodialysis  Conventional treatments (including haemodialysis, haemoperfusion, calamine lotion, and cetirizine) | Uremic pruritus |
| 119 | Oral Chinese Herbal Medicine for Depressive Disorder in Patients after Percutaneous Coronary Intervention: A Systematic Review and Meta-Analysis | Chinese herbal formulae (including shugan jieyu capsule, jieyu granules, shugan jieyu decoction, danlou tablet, jieyu tongmai decoction, bushenningxin decoction, jieyu tongbi decoction, shugan jieyu anshen cream, jieyu anshen decocotion, yangxin jieyu decoction, and xinkeshu tablet)  Chinese herbal formulae + Coventional treatments (including citalopram, flupentixol, melitracen, and paroxetine) | Placebo  Routine care  Coventional treatments (including citalopram, flupentixol, melitracen, and paroxetine) | Depression |
| 120 | Chinese medicinal herbs in the treatment of diabetic cognitive impairment: A systematic review and meta-analysis | Chinese herbal formulae (including yishen huoxue formula, bushen quyu yizhi decoction, bushen huoxue kaiqiao formula, zhinao granules, bushen quyu yizhi decoction, jinmaitong, tangnaoqing granules, bushen jiannao formula, and bushen huoxue kaiqiao formula)  Chinese herbal formulae + Conventional treatments (including nimodipine and aspirin) | Conventional treatments (including nimodipine, aspirin, and huperzine A) | Diabetic cognitive impairment |
| 121 | Acupuncture plus Chinese Herbal Medicine for Irritable Bowel Syndrome with Diarrhea: A Systematic Review and Meta-Analysis | Chinese herbal formulae (including modified sini decoction, tongxie yaofang, modified chaishao yigong and xiangsha pingwei decoction, chang ling capsule, huoxiang zhengqi pill, jianpi huazhuo tongluo decoction, chaihu shugan decoction, modified xiaoyaosan, dunhuang baoyuan decoction, banxia xiexin decoction, shugan jianpi zhixie decoction, and modified shenling baizhu powder) + Acupuncture | Conventional treatments (including belladonna tablet, combined bifidobacterium lactobacillus enterococcus and bacillus cereus tablet, compound glutamine enteric-coated capsule, diazepam, live bifidobacterium preparation, loperamide, montmorillonite, otilonium bromide, andyzanol, pinaverium bromide, and saccharomyces boulardii sachets) | Diarrhoea predominant irritable bowel syndrome |
| 122 | Chinese herbal medicine for diabetic kidney disease: A systematic review and meta-analysis of randomised placebo-controlled trials | Chinese herbal formulae (including qikui granules, qiwei granules, arctiin granules, xuezhikang capsule, gandi capsue, liuweidihuang pill, ginkgo biloba tablet, qiming granules, qiwei granules, tangshen granules, qihuang capsule, qiyao xiaoke capsule, qizhu granules, tangshenning granules, baoshen pill, sanhuang yishen granules, and tangweikang capsule)  Chinese herbal formulae + Conventional treatments (including benazepril, losartan, tripterygium glycosides, and irbesartan) | Placebo  Conventional treatments (including losartan, benazepril, irbesartan, valsartan, and captopril) | Diabetic kidney disease |
| 123 | Managing Depression with Bupleurum chinense Herbal Formula: A Systematic Review and Meta-Analysis of Randomized Controlled Trials | Bupleurum chinense preparations (including xiaoyao powder, chaihushugan powder, suanzaoren decoction, and bushen shugan huayu decoction) | Convention treatments (including fluoxetine, paroxetine, sertraline, citalopram, and escitalopram) | Depression |
| 124 | Efficacy and Safety of Fuzi Formulae on the Treatment of Heart Failure as Complementary Therapy: A Systematic Review and Meta-Analysis of High-Quality Randomized Controlled Trials | Aconitum carmichaelii preparations (including huaxinsu granules, nuanxin capsule, tongyang huoxue decoction, kangshuai decoction, qili qiangxin capsule, shenfu cardiac pill, baoyuan shipi decoction, and shenfu jiuxin decoction) + Conventional treatments (not specified) | Conventional treatments (not specified) | Heart failure |
| 125 | External application of Ruyi Jinhuang powder for phlebitis: A systematic review and meta-analysis | Ruyi jinhuang powder | 50% Magnemsulphate  Hirudoid cream | Phlebitis |
| 126 | The effect of Chinese herbal medicine on quality of life and exercise tolerance in heart failure with preserved ejection fraction: A systematic review and meta-analysis of randomized controlled trials | Chinese herbal formulae (including qili qiangxin capsule, yiqi yangyin huoxue decoction, bawei tongluo granules, tongmai baoxin decoction, huoxue lishui decoction, jiawei zhenwu decoction, wenxin granules, shenmai injection, danshen chuanxiongqin injection, shuxuetong injection, and yixinshu capsule) + Conventional treatments (including benazepril, metoprolol, imidapril, bisoprolol, aldactone, isosorbide, and enalapril) | Conventional treatments (including benazepril, metoprolol, imidapril, bisoprolol, aldactone, isosorbide, and enalapril) | Heart failure with preserved ejection fraction |
| 127 | Tian Wang Bu Xin Dan for Insomnia: A Systematic Review of Efficacy and Safety | Tianwangbuxindan  Tianwangbuxindan + Conventional treatments (including diazepam and estazolam) | Conventional treatments (including estazolam, bailemian capsule, andyzanol, alprazolam, and diazepam) | Insomnia |
| 128 | Chinese herbal medicine Dengzhan Shengmai capsule as adjunctive treatment for ischemic stroke: A systematic review and meta-analysis of randomized clinical trials | Dengzhan shengmai capsule + Conventional treatments (not specified) | Conventional treatments (not specified) | Ischemic stroke |
| 129 | Effects of adding Rheum officinale to angiotensin-converting enzyme inhibitors or angiotensin receptor blockers on renal function in patients with chronic renal failure: A meta-analysis of randomized controlled trials | Rheum officinale preparations (including uremic clearance granules, shenshuaining capsule, and xinqingning tablet) + Conventional treatments (including enalapril, irbesartan, telmisartan, losartan, benazepril, and valsartan) | Conventional treatments (including enalapril, irbesartan, telmisartan, losartan, benazepril, and valsartan) | Chronic renal failure |
| 130 | Motherwort injection for preventing postpartum hemorrhage in women with vaginal delivery: A systematic review and meta-analysis of randomized evidence | Motherwort injection  Motherwort injection + Oxytocin | Oxytocin | Postpartum haemorrhage |
| 131 | Effectiveness and Safety of Oral Cordyceps sinensis on Stable COPD of GOLD Stages 2-3: Systematic Review and Meta-Analysis | Cordyceps sinensis preparations (including bailing capsule, hechechongcao capsule, and jinshuibao capsule)  Cordyceps sinensis preparations + Conventional treatments (including budesonide/formoterol, salmeterol/fluticasone, aminophylline, and ambroxol hydrochloride) + Routine care | Conventional treatments (including budesonide/formoterol, salmeterol/fluticasone, aminophylline, and ambroxol hydrochloride) + Routine care | Chronic obstructive pulmonary disease |
| 132 | Cinobufotalin injection combined with chemotherapy for the treatment of advanced NSCLC in China: A PRISMA-compliant meta-analysis of 29 randomized controlled trials | Cinobufotalin injection + Conventional treatments (including cisplatin, docetaxel, etoposide, gemcitabine, ifosfamide, navelbine, navelbine, paclitaxel, carboplatin, pemetrexed, and vindesine) | Conventional treatments (including cisplatin, docetaxel, etoposide, gemcitabine, ifosfamide, navelbine, navelbine, paclitaxel, carboplatin, pemetrexed, and vindesine) | Advanced non-small-cell lung cancer |
| 133 | Effectiveness of Chinese herbal medicine for patients with primary insomnia: A PRISMA-compliant meta-analysis | Chinese herbal formulae (including suanzaoren gao, huolisu koufuye, yehejiannao tablet, zaorenanshen capsule, jiaweixiaoyao powder, chanyeanshen capsule, wuling capsule, sanqi granules, bailemian capsule, nanwuweizi capsule, xinrenshenan capsule, zaorenanshen tablet. xie cao, meian granules, and haishe capsule) | Placebo | Primary insomnia |
| 134 | Efficacy and safety of naoshuantong capsule in the treatment of ischemic stroke: A meta-analysis | Naoshuantong capsule + Conventional treatments (including aspirin, clopidogrel, citicoline, atorvastatin, and breviscapine) | Conventional treatments (including aspirin, clopidogrel, citicoline, simvastatin, atorvastatin, and breviscapine) | Ischemic stroke |
| 135 | Traditional Chinese medicine for coronary heart disease: Clinical evidence and possible mechanisms | Chinese herbal forlumae (including xiongshao capsule, tongguan capsule, xuezhikang capsule, qingre quyu granules, compound salvia tablet and xinyue capsule, shenshao tablet, xuefu zhuyu capsule, tongxinluo, chuangxiongol, yixinmai granules, betel, double ginseng capsule and tongguan capsule, chekshincentongxin granules, musk baoxin pill, shenzhu guanxin formula, wufuxinnaoqing capsule, live heart pill, danlou tablet, xuesaitong soft capsule, and coronary ningtong prescription) + Conventional treatments (including clopidogrel, aspirin, atorvastatin, low molecular weight heparin, bisoprolol, isosorbide dinitrate sustained release tablet, and thiazepine) | Placebo | Coronary heart disease |
| 136 | Chinese herbal medicine formulas as adjuvant therapy for osteonecrosis of the femoral head: A systematic review and meta-analysis of randomized controlled trials | Chinese herbal formulae (including bushenhuoxue decoction, huoxuejiangu decoction, guhuaisi No funding.2 decoction, jianbuhuqian pill, luguishenggu pill, sijunzi decoction, taohongsiwu decoction, huoluogukang pill, wentonghuoxue decoction, yiqihuoxuebushentongluo decoction, and jiangu decoction) + Core decompression | Core decompression | Osteonecrosis of the femoral head |
| 137 | Efficacy of the Oriental herbal medicine, Jie Yu Dan, for alleviating post-stroke aphasia: A Systematic Review and meta-analysis of randomized clinical trials | Jie Yu Dan  Jie Yu Dan + Symptomatic treatments and interventions | Conventional treatments (including acupuncture, ozagrel sodium, and erigeron, and other symptomatic treatments and interventions) | Post-stroke aphasia |
| 138 | Chinese Herbal Medicine for Advanced Non-Small-Cell Lung Cancer: A Systematic Review and Meta-Analysis | Chinese herbal medicine (not specified)  Chinese herbal medicine (not specified) + Gefitinib | Gefitinib, erlotinib, and icotinib | Advanced non-small-cell lung cancer |
| 139 | Chinese herbal medicines on cognitive function and activity of daily living in senior adults with Alzheimer's disease: a systematic review and meta-analysis | Chinese herbal formulae (including qingxin yizhi decoction, bushenhuatanyizhi instant granules, congrong yizhi decoction, yizhi jiannao granules, shenghuang yizhi decoction, danggui shaoyao powder, rehmanniae decoction, tongqiaohuoxue decoction, jiaweizuogui pill, yishen huazhuo granules, bushen formula, yizhi jiannao granules, buyanghuanwu decoction, reinhartdt and sea cucumber capsule, refined xingnao powder, nailing decoction, tiaoxin formula, wenpitongluokaiqiao decoction, yizhijiannao granules, yizhi decoction, and zhijingkoufuye) | Conventional treatments (including donepezil, piracetam, nimodipine, and sanlexi) | Alzheimer's disease |
| 140 | The therapeutic effect of Chaihu-Shugan-San in fatty liver disease: A meta-analysis randomized controlled trials | Chaihu shugan powder | Conventional treatments (including dongbao liver thai, polyene phosphatidyl choline, fenofibrate sustained release capsule, and zhibituo jujue tablet) + Routine treatments (including health education, alcohol consumption and diet control, and sports) | Fatty liver disease |
| 141 | A Review of Danshen Combined with Clopidogrel in the Treatment of Coronary Heart Disease | Salvia Miltiorrhiza preparations (including compound danshen dropping pills, danshen and ligustrazine hydrochloride Injection, and guanxin danshen dropping pill) | Clopidogrel | Coronary heart disease |
| 142 | The Therapeutic Effects of a Traditional Chinese Medicine Formula Wuzi Yanzong Pill for the Treatment of Oligoasthenozoospermia: A Meta-Analysis of Randomized Controlled Trials | Wuzi Yanzong Pill | Placebo  Vitamin control  Kidney essence granules  Gonadotropins | Oligo-asthenozoospermia |
| 143 | Efficacy of Chinese herbal medicine in treatment of allergic rhinitis in children: a meta-analysis of 19 randomized controlled trials | Chinese herbal formulae (including suhuang zhike capsule, bimin powder, wenfei zhiliu dan, and yupingfeng granules) | Conventional treatments (including loratadine, montelukast sodium, cetirizine dihydrochloride, dermatophagoides farinae, budesonide, prednisone acetate, ketotifen fumarate, ephedrine hydrochloride, nitrofurazone, terfenadine, and biyankang) | Allergic rhinitis |
| 144 | Suanzaoren formulae for insomnia: Updated clinical evidence and possible mechanisms | Semen Ziziphi Spinosae preparations (including tangyushu powder, mei'an capsule, fufanganmei decoction, xinshenning tablet, fuzhazaoren decoction, banbaizhenhun decoction, zaoren'anshen tablet, jiawei xiaoyao powder, shen'an III capsule, chanyeanshen capsule, qingxinzishui capsule, and sanqi granules) | Placebo  Conventional treatments (including estazolam, alprazolam, and oryzanol) | Insomnia |
| 145 | Clinical Effects and Safety of Tongxieyaofang on Diarrhea Predominant Irritable Bowel Syndrome: A Meta-Analysis of Randomized Trails | Tongxieyaofang | Placebo  Conventional treatments (including dioctahedral smectite, pinaverium bromide, andyzanol, trimebutine, bifid triple viable capsule, bacillus subtilis and enterococcus bacteria capsule, montmorillonite, loperamide, otilonium bromide, dioctahedral smectite, vitamin K, and bifidobacterium and lactobacillus tablet) | Diarrhoea predominant irritable bowel syndrome |
| 146 | Effects of Ginger (Zingiber officinale Roscoe) on Type 2 Diabetes Mellitus and Components of the Metabolic Syndrome: A Systematic Review and Meta-Analysis of Randomized Controlled Trials | Ginger | Placebo | Type 2 diabetes |
| 147 | Traditional Chinese and western medicine for the prevention of deep venous thrombosis after lower extremity orthopedic surgery: a meta-analysis of randomized controlled trials | Chinese herbal formulae (including yiqi poxue prescriptions, taohong siwu soup, ligustrazine hydrochloride, yiqi huoxue huayu soup, danshen injection liquid, gubu soup, huoxue tongmai soup, danggui shaoyao powder, buyang huanwu soup, shuxue tongmai injection liquid, huoxue zhuyu tongluo soup, and honghua injection liquid) | Conventional treatments (including low molecular weight heparin, rivaroxaban, and aspirin) | Deep venous thrombosis |
| 148 | Therapeutic Efficacy of Kangfuxin Liquid Combined with PPIs in Gastric Ulcer | Kangfuxin liquid + Proton pump inhibitor (including esomeprazole, lansoprazole, rabeprazole, and pantoprazole) | Proton pump inhibitors (including esomeprazole, lansoprazole, rabeprazole, and pantoprazole) | Gastric ulcer |

**Table S5.** List of excluded systematic reviews on Chinese herbal medicine after assessing full text for eligibility and reasons for exclusion

| **Excluded systematic reviews** | **Reasons** |
| --- | --- |
| 1. Adams D, Wu T, Yang X, Tai S, Vohra S. Traditional Chinese medicinal herbs for the treatment of idiopathic chronic fatigue and chronic fatigue syndrome. Cochrane Database of Systematic Reviews. 2009(4). | Withdrawal from publication |
| 1. Ainehchi N, Farshbaf-Khalili A, Ghasemzadeh A, Hamdi K, Khaki A, Ouladsahebmadarek E, Delazar A, Bakhtyari F, Mazandarani M. The effect of herbal medicine supplementation on clinical and para-clinical outcomes in women with PCOS: A systematic review and meta-analysis. International Journal of Women's Health and Reproduction Sciences. 2019;7(4):423-433. | Not evaluating CHM |
| 1. Appleton L, Day AS. Re: A Meta-Analysis of the Clinical Use of Curcumin for Irritable Bowel Syndrome. | Not MA |
| 1. Bu F, Wang W, Chen R, Lin Z, Han M, Robinson N, Liu X, Lu C, Liu J. Adding Chinese herbal medicine to probiotics for irritable bowel syndrome-diarrhea: A systematic review and meta-analysis of randomized controlled trials. Journal of Traditional Chinese Medical Sciences. 2020 Mar 1;7(1):20-36. | Not evaluating CHM |
| 1. Chen R, Xiao Y, Chen M, He J, Huang M, Hong X, Liu X, Fu T, Zhang J, Chen L. A traditional Chinese medicine therapy for coronary heart disease after percutaneous coronary intervention: a meta-analysis of randomized, double-blind, placebo-controlled trials. Bioscience reports. 2018 Oct 31;38(5). | Not evaluating CHM |
| 1. Chen Y, Zheng Y, Shergis JL, Wu L, Zhang AL, Guo X, Lin L, Xue CC, Xu Y. Acupoint application of herbal medicine combined with pharmacotherapy for stable asthma: a meta-analysis. Advances in Integrative Medicine. 2019 May 1;6:S60. | Not evaluating CHM |
| 1. Dai L, Chen L, Wang W. Safety and Efficacy of Saffron (Crocus sativus L.) for Treating Mild to Moderate Depression: A Systematic Review and Meta-analysis. The Journal of nervous and mental disease. 2020 Apr 1;208(4):269-76. | Not evaluating CHM |
| 1. Deyno S, Eneyew K, Seyfe S, Tuyiringire N, Peter EL, Muluye RA, Tolo CU, Ogwang PE. Efficacy and safety of cinnamon in type 2 diabetes mellitus and pre-diabetes patients: A meta-analysis and meta-regression. diabetes research and clinical practice. 2019 Oct 1;156:107815. | Not evaluating CHM |
| 1. Ghaderi A, Asbaghi O, Reiner Ž, Kolahdooz F, Amirani E, Mirzaei H, Banafshe HR, Dana PM, Asemi Z. The effects of saffron (Crocus sativus L.) on mental health parameters and C-reactive protein: A meta-analysis of randomized clinical trials. Complementary Therapies in Medicine. 2020 Jan 1;48:102250. | Not evaluating CHM |
| 1. Ghorbani Z, Mirghafourvand M. A Meta-Analysis of the Efficacy of Panax Ginseng on Menopausal Women’s Sexual Function. International Journal of Womens Health and Reproduction Sciences. 2019 Jan 1;7(1):124-33. | Not evaluating CHM |
| 1. Guo J, Li B, Wu W, Wang Z, Wang F, Guo T. Chinese herbal medicines compared with n-acetylcysteine for the treatment of idiopathic pulmonary fibrosis: a systematic review of randomized controlled trials. Evidence-Based Complementary and Alternative Medicine. 2019 Jun 1;2019. | Not evaluating CHM |
| 1. Hernández-García D, Granado-Serrano AB, Martín-Gari M, Naudí A, Serrano JC. Efficacy of Panax ginseng supplementation on blood lipid profile. A meta-analysis and systematic review of clinical randomized trials. Journal of ethnopharmacology. 2019 Oct 28;243:112090. | Not evaluating CHM |
| 1. Kachura A, Lee T, Sanchez ML, Rhéaume É, Zaghrini W, Liu R, Haddad PS, Overy DP, Harris CS. Yarrow by Parts: An Ethnobotanical, Pharmacological, and Metabolomics Analysis of One of North America's Most Important Medicinal Plants. Planta Medica International Open. 2018 May;5(S 01):Pharm02. | Conference abstract |
| 1. Ko SJ, Park JW, Lee J, Lee JH, Cho SH, Kuo B, Nam S, Ha N, Kim J. Tu1587-Efficacy and Safety of Yukgunja-Tang for the Treatment of Functional Dyspepsia: Systematic Review and Meta-Analysis. Gastroenterology. 2018 May 1;154(6):S-958. | Conference abstract |
| 1. Lin S, An X, Guo Y, Jin H, Xie T, Sui X. Meta-analysis of Astragalus-containing Traditional Chinese Medicine Combined with Chemotherapy for Colorectal cancer. Frontiers in oncology. 2019;9:749. | Not evaluating CHM |
| 1. Maharlouei N, Tabrizi R, Lankarani KB, Rezaianzadeh A, Akbari M, Kolahdooz F, Rahimi M, Keneshlou F, Asemi Z. The effects of ginger intake on weight loss and metabolic profiles among overweight and obese subjects: A systematic review and meta-analysis of randomized controlled trials. Critical reviews in food science and nutrition. 2019 Jun 17;59(11):1753-66. | Not evaluating CHM |
| 1. Marx W, Lane M, Rocks T, Ruusunen A, Loughman A, Lopresti A, Marshall S, Berk M, Jacka F, Dean OM. Effect of saffron supplementation on symptoms of depression and anxiety: a systematic review and meta-analysis. Nutrition reviews. 2019 Aug 1;77(8):557-71. | Not evaluating CHM |
| 1. Namazi N, Khodamoradi K, Khamechi SP, Heshmati J, Ayati MH, Larijani B. The impact of cinnamon on anthropometric indices and glycemic status in patients with type 2 diabetes: A systematic review and meta-analysis of clinical trials. Complementary therapies in medicine. 2019 Apr 1;43:92-101. | Not evaluating CHM |
| 1. Rahmani J, Bazmi E, Clark C, Nazari SS. The effect of Saffron supplementation on waist circumference, HA1C, and glucose metabolism: A systematic review and meta-analysis of randomized clinical trials. Complementary Therapies in Medicine. 2020 Mar 1;49:102298. | Not evaluating CHM |
| 1. Rahmani J, Manzari N, Thompson J, Clark CC, Villanueva G, Varkaneh HK, Mirmiran P. The effect of saffron on weight and lipid profile: A systematic review, meta‐analysis, and dose–response of randomized clinical trials. Phytotherapy research. 2019 Sep;33(9):2244-55. | Not evaluating CHM |
| 1. Shi X, Zhong X, Ding J. Adjuvant treatment with Yupingfeng formula for primary nephrotic syndrome in children: A PRISMA systematic review and meta-analysis of randomized controlled trials. Medicine. 2018 Jul;97(29). | Not evaluating CHM |
| 1. Toth B, Nemeth D, Soos A, Hegyi P, Pham-Dobor G, Varga O, Varga V, Kiss T, Sarlos P, Eross B, Csupor D. The Effects of a Fixed Combination of Berberis aristata and Silybum marianum on Dyslipidaemia - A Meta-analysis and Systematic Review. Planta Medica. 2020;86(2):132-143. | Not evaluating CHM |
| 1. Vallianou N, Tsang C, Taghizadeh M, Davoodvandi A, Jafarnejad S. Effect of cinnamon (Cinnamomum Zeylanicum) supplementation on serum C-reactive protein concentrations: A meta-analysis and systematic review. Complementary Therapies in Medicine. 2019 Feb 1;42:271-8. | Not evaluating CHM |
| 1. Wen L, Tian X, Wang D, Xia R, Fei Y, Huang N, Hu R, Liu J. Patent of ophiocordyceps sinensis (Jin Shui Bao) for diabetic kidney disease: A systematic review and meta-analysis. Global Advances in Health and Medicine. 2018;7(5):197-199. | Conference abstract |
| 1. Xu L, Xu XY, Hou XQ, Wang FG, Gao S, Zhang HT. Adjuvant therapy with Astragalus membranaceus for post-stroke fatigue: a systematic review. Metabolic brain disease. 2020 Jan 1:1-1. | Not evaluating CHM |
| 1. Xu QQ, Shan CS, Wang Y, Shi YH, Zhang QH, Zheng GQ. Chinese herbal medicine for vascular dementia: a systematic review and meta-analysis of high-quality randomized controlled trials. Journal of Alzheimer's Disease. 2018 Jan 1;62(1):429-56. | Not evaluating CHM |
| 1. Yang L, Di YM, Shergis JL, Li Y, Zhang AL, Lu C, Guo X, Xue CC. A systematic review of acupuncture and Chinese herbal medicine for postpartum depression. Complementary Therapies in Clinical Practice. 2018 Nov 1;33:85-92. | Not evaluating CHM |
| 1. Yang L, Shergis J, Di Y, Zhang A, Lu C, Guo X, Li Y, Xue C. A systematic review of Chinese herbal medicine for postpartum depression. Advances in Integrative Medicine. 2019;6 (Supplement 1):S58. | Conference abstract |
| 1. Yang L, Zhang L, Shergis J, Mao W, Zhang A, Liu X, Lu C, Guo X, Xue C. A systematic review of outcomes reported in randomised controlled trials evaluating Chinese herbal medicine for diabetic kidney disease. 2019;6 (Supplement 1): S82-S83. | Conference abstract |
| 1. Yoshino T, Arita R, Horiba Y, Watanabe K. The use of maoto (Ma-Huang-Tang), a traditional Japanese Kampo medicine, to alleviate flu symptoms: a systematic review and meta-analysis. BMC complementary and alternative medicine. 2019 Dec 1;19(1):68. | Not evaluating CHM |
| 1. Zhang L, Shergis JL, Yang L, Zhang AL, Guo X, Zhang L, Zhou S, Zeng L, Mao W, Xue CC. Astragalus membranaceus (Huang Qi) as adjunctive therapy for diabetic kidney disease: an updated systematic review and meta-analysis. Journal of ethnopharmacology. 2019 Jul 15;239:111921. | Not evaluating CHM |
| 1. Zhang L, Shergis JL, Yang L, Zhang AL, Guo X, Zhang L, Zhou S, Zeng L, Mao W, Xue CC. Astragalus membranaceus (Huang Qi) as adjunctive therapy for diabetic kidney disease: an updated systematic review and meta-analysis. Journal of ethnopharmacology. 2019 Jul 15;239:111921. | Not evaluating CHM |
| 1. Zhang X, Lan F, Zhang Y, Zhang L. Chinese herbal medicine to treat allergic rhinitis: evidence from a meta-analysis. Allergy, asthma & immunology research. 2018 Jan 1;10(1):34-42. | Not evaluating CHM |
| 1. ZHAO J, LIAO X, ZHAO H, LI ZG, WANG NY, WANG LM. Evaluation on Effectiveness and Safety of Chinese Herbs inTreatment of Sub-health: A Systematic Review andMeta-Analysis of Randomized Controlled Trials. Chinese Journal of Integrative Medicine. 2019(6):11. | Not evaluating CHM |
| 1. Ziaei R, Foshati S, Hadi A, Kermani MA, Ghavami A, Clark CC, Tarrahi MJ. The effect of nettle (Urtica dioica) supplementation on the glycemic control of patients with type 2 diabetes mellitus: A systematic review and meta‐analysis. Phytotherapy Research. 2020 Feb;34(2):282-94. | Not evaluating CHM |

*Keys:* CHM: Chinese herbal medicine. MA: Meta-analysis.
